# Anticancer Effects of Cucurbitacin B and Meleagrin Associated with TYRO3 Downregulation in Colorectal Cancer Cells

**DOI:** 10.3390/ijms27156759

**Published:** 2026-07-28

**Authors:** Reha Sertac Ilhan, Merve Gurboga, Turgut Sekerler, Pinar Ulupinar, Derya Ozsavci, Ozlem Bingol Ozakpinar

**Affiliations:** Department of Biochemistry, Faculty of Pharmacy, Marmara University, Istanbul 34854, Türkiye; merve.gurboga@marmara.edu.tr (M.G.); turgut.sekerler@marmara.edu.tr (T.S.); pinar.ulupinar@marmara.edu.tr (P.U.); derya.ozsavci@marmara.edu.tr (D.O.); ozlem.bingol@marmara.edu.tr (O.B.O.)

**Keywords:** colorectal cancer, TYRO3, Cucurbitacin B, meleagrin, apoptosis, receptor expression

## Abstract

Colorectal cancer (CRC) remains a major cause of cancer mortality worldwide, highlighting the need for novel molecular targets and alternative therapeutic strategies. TYRO3, a member of the TAM receptor tyrosine kinase family, has been associated with tumor progression and poor prognosis in CRC. In this study, the effects of the natural compounds Meleagrin and Cucurbitacin B on TYRO3 expression and CRC cell behavior were investigated in HCT-116 and HT-29 cells. Cell proliferation, apoptosis, migration, and TYRO3 expression were evaluated using functional and expression-based analyses. Both compounds modulated TYRO3 expression and suppressed proliferation and wound closure dynamics in CRC cells. Cucurbitacin B exerted more pronounced antiproliferative and pro-apoptotic effects, whereas Meleagrin demonstrated antiproliferative activity with comparatively lower effects on normal colon epithelial cells (CCD 841 CoN), suggesting a potentially more favorable selectivity profile. Collectively, these findings support further mechanistic investigation of TYRO3-modulating natural compounds as potential therapeutic candidates for CRC.

## 1. Introduction

Colorectal cancer (CRC) remains one of the leading causes of cancer-related morbidity and mortality worldwide, ranking as the third most commonly diagnosed cancer and the second leading cause of cancer-related deaths [1]. Despite significant advances in surgical techniques, chemotherapy, targeted therapies, and immunotherapy, the prognosis of patients with advanced-stage CRC remains poor, largely due to late-stage diagnosis and metastatic dissemination. Approximately 20–25% of CRC patients present with metastatic disease at the time of diagnosis, while nearly half eventually develop metastases during disease progression [2]. Consequently, 5-year survival rates in metastatic CRC remain typically below 20% [3,4]. In addition, the pronounced molecular heterogeneity of CRC further complicates treatment efficacy and limits the success of existing therapeutic strategies [5]. Recent advances in molecular classification and precision oncology have further underscored the biological complexity of CRC, demonstrating that distinct molecular subtypes are associated with marked differences in tumor biology, therapeutic response, and clinical outcome [6,7,8]. Collectively, these observations underscore the need to identify novel molecular targets and develop more effective targeted therapeutic strategies to improve patient outcomes.

The TAM receptor family, consisting of TYRO3, AXL, and MERTK, has emerged as an important regulator of tumor progression, immune modulation and therapeutic resistance in various cancers [5,9,10,11]. Although all three receptors have been implicated in CRC biology, accumulating evidence suggests that their biological functions and therapeutic relevance differ substantially [10,11]. Although AXL has been the most extensively investigated TAM receptor in CRC, increasing evidence suggests that TYRO3 represents a comparatively underexplored therapeutic target despite its emerging oncogenic relevance. AXL has been associated with epithelial–mesenchymal transition (EMT), migration, invasion, metastatic progression, and resistance to anti-epidermal growth factor receptor (EGFR) therapies [10,11,12,13,14,15,16]. However, its prognostic and functional significance in CRC remains inconsistent across studies, with variable associations reported between AXL expression and patient survival or disease progression [12,13,14,15]. In contrast, MERTK has been linked predominantly to immune regulation, macrophage-mediated signaling, and intestinal inflammatory homeostasis rather than direct promotion of aggressive tumor behavior [17,18]. Experimental evidence further demonstrated that simultaneous loss of AXL and MERTK signaling may exacerbate inflammation-associated colon tumorigenesis, highlighting the context-dependent roles of TAM receptors within the intestinal tumor microenvironment [18].

In contrast, accumulating evidence suggests that TYRO3 is more consistently associated with aggressive CRC biology and disease progression. TYRO3 is significantly overexpressed in primary CRC tissues, precancerous polyps, and metastatic lesions compared with normal colonic mucosa, and increased TYRO3 expression has been associated with advanced TNM stage, lymph node metastasis, neural invasion, disease progression, and poor overall survival in CRC patients [19,20,21,22,23,24]. Beyond these effects, experimental and clinicopathological studies demonstrated that aberrant TYRO3 expression occurs during early stages of colorectal tumorigenesis and progressively increases with disease advancement, suggesting a potential role in both tumor initiation and progression [20,21]. Functional studies demonstrated that TYRO3 contributes to CRC cell proliferation, migration, invasion, EMT, metastatic dissemination, and tumor cell survival through activation of oncogenic signaling pathways, including phosphoinositide 3-kinase/protein kinase B (PI3K/AKT), mitogen-activated protein kinase/extracellular signal-regulated kinase (MAPK/ERK), and signal transducer and activator of transcription 3 (STAT3) signaling networks, and *SNAI1*-associated signaling networks [19,23,24,25]. Moreover, TYRO3 has increasingly been implicated in therapy resistance, as its expression is elevated in drug-resistant CRC cells and its inhibition restores sensitivity to 5-fluorouracil while suppressing proliferative and migratory capacities [22]. Pharmacological or genetic suppression of TYRO3 has also been shown to reduce tumor growth, induce apoptosis, attenuate invasive phenotypes, and enhance responsiveness to anticancer therapies in gastrointestinal cancer models [19,26,27]. Collectively, these findings indicate that TYRO3 represents a promising yet relatively underexplored molecular target in CRC and further support the investigation of TYRO3-directed therapeutic strategies [24,28].

Natural compounds have attracted increasing attention as potential anticancer agents because of their structural diversity, ability to modulate multiple tumor-promoting signaling pathways, and relatively favorable pharmacological profiles compared with conventional chemotherapeutic agents [29,30,31]. Recent evidence further highlights natural products as an important source of multi-target anticancer agents capable of simultaneously regulating cell proliferation, apoptosis, migration, invasion, angiogenesis, and therapeutic resistance through modulation of multiple oncogenic signaling pathways. Moreover, recent comprehensive reviews have emphasized their growing potential as lead compounds for targeted drug discovery and preclinical colorectal cancer therapy, while also highlighting the importance of mechanistic validation and translational development [32,33]. In particular, natural-product-derived compounds have emerged as promising sources of kinase-targeting agents capable of interfering with tumor proliferation, survival, migration, and therapeutic resistance pathways.

To identify natural products with potential TYRO3-modulating activity, an initial structure-based virtual screening of a natural compound library was performed using the iGEMDOCK platform. Candidate compounds were prioritized according to their predicted TYRO3-binding affinities. Among the highest-ranked candidates, Cucurbitacin B and Meleagrin were selected for experimental validation because they combined favorable predicted TYRO3-binding affinity with complementary biological characteristics, including previously reported anticancer activity, complementary chemical scaffolds, and practical suitability for experimental validation. Thus, the selection of these compounds was guided primarily by the virtual screening results and further supported by their reported biological properties and experimental feasibility.

Meleagrin, an indole alkaloid derived from *Penicillium* species, has demonstrated multitarget anticancer activity through modulation of several cancer-associated kinases and signaling proteins [34,35]. Earlier investigations demonstrated that Meleagrin exerts antiproliferative and pro-apoptotic effects, suppresses migration and invasion, and induces G2/M cell-cycle arrest in different tumor models [34,35,36]. Importantly, Meleagrin was previously identified as an inhibitor of c-Met-dependent breast cancer progression, supporting its ability to interfere with kinase-driven oncogenic signaling [35]. Similarly, Cucurbitacin B, a tetracyclic triterpenoid compound derived from plants of the Cucurbitaceae family, has been extensively associated with broad-spectrum anticancer activity through inhibition of proliferation, induction of apoptosis, suppression of migration and invasion, and modulation of multiple oncogenic signaling pathways involved in tumor growth and survival [37,38]. In CRC models, CuB was shown to induce G2/M cell-cycle arrest, activate caspase-dependent apoptosis, suppress cancer stemness, and inhibit metastatic phenotypes [38,39,40]. Furthermore, Cucurbitacin B has been reported to modulate the tumor microenvironment by suppressing M2-like macrophage polarization and attenuating metastatic progression in CRC models [40]. Therefore, the combination of favorable virtual screening results, previously reported kinase-targeting anticancer properties, and practical suitability for experimental validation provided a strong scientific rationale for selecting Cucurbitacin B and Meleagrin for the present study.

Importantly, available preclinical evidence further supports the investigation of these compounds from a translational perspective. Meleagrin has previously demonstrated significant antitumor efficacy in orthotopic xenograft models without apparent systemic toxicity or significant body weight loss, suggesting a favorable preliminary safety profile [35]. Likewise, Cucurbitacin B has shown robust antitumor activity in multiple murine colorectal cancer models, where treatment effectively suppressed tumor growth while exhibiting no significant body weight loss or detectable histopathological toxicity in major organs under the investigated experimental conditions [38,40,41]. Furthermore, recent comprehensive reviews have highlighted that although Cucurbitacin B exhibits dose-dependent toxicity, optimization strategies including structural modification, combination therapy, and nanoformulation approaches may substantially improve its therapeutic index and translational potential [42]. Collectively, these findings provide both a mechanistic and translational rationale for selecting Cucurbitacin B and Meleagrin as candidate TYRO3-modulating compounds for colorectal cancer.

Despite increasing evidence supporting TYRO3 as a therapeutic target in CRC, no previous study has investigated whether Cucurbitacin B or Meleagrin modulate TYRO3 expression and associated cellular responses in colorectal cancer. Therefore, this study aimed to investigate the anticancer effects of Cucurbitacin B and Meleagrin in CRC cells and to determine whether these effects are associated with modulation of TYRO3 expression by integrating proliferation, apoptosis, migration, and gene/protein expression analyses.

## 2. Results

### 2.1. TYRO3 Expression in CRC Cell Lines

**Figure 1:**
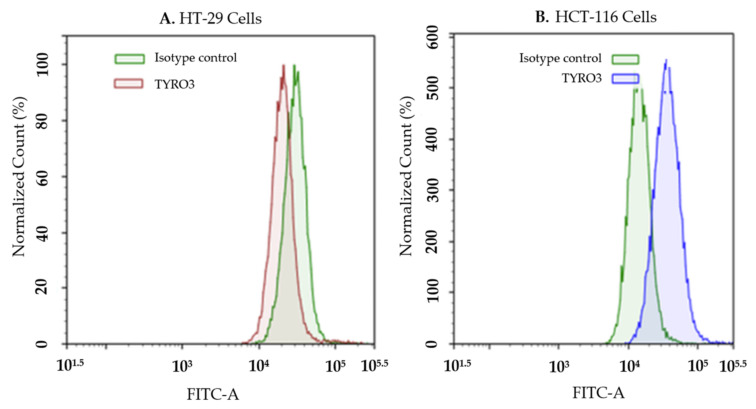
Basal TYRO3 expression in CRC cell lines determined by flow cytometry. Representative overlaid histograms showing TYRO3 fluorescence intensity in HT-29 (**A**) and HCT-116 (**B**) CRC cells relative to the isotype control. Fluorescence intensity is presented as normalized cell count (%) versus FITC-A fluorescence intensity. HCT-116 cells exhibited a more evident rightward fluorescence shift compared with HT-29 cells, indicating relatively higher basal TYRO3 expression.

Basal TYRO3 protein expression was evaluated in HT-29 and HCT-116 CRC cell lines using flow cytometry (Figure 1). Representative histograms demonstrated minimal separation between TYRO3 and isotype control signals in HT-29 cells, indicating relatively low basal TYRO3 expression. In contrast, HCT-116 cells exhibited a more evident rightward shift in fluorescence intensity relative to the isotype control, suggesting higher TYRO3-associated fluorescence and increased basal TYRO3 expression. Overall, comparative analysis of fluorescence profiles revealed clear cell line-dependent differences in TYRO3 expression between HT-29 and HCT-116 CRC cells.

### 2.2. Effects of Meleagrin and Cucurbitacin B on TYRO3 Expression

#### 2.2.1. Flow Cytometric Analysis of TYRO3 Protein Expression

**Figure 2:**
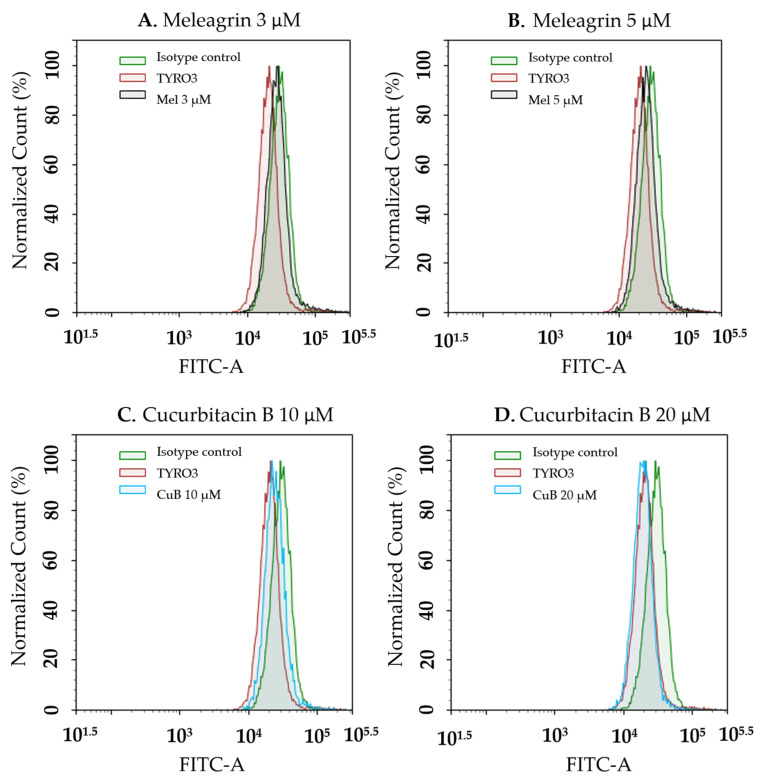
Flow cytometric analysis of TYRO3 fluorescence profile in HT-29 CRC cells following treatment with Meleagrin and Cucurbitacin B. Representative overlaid histograms showing TYRO3 fluorescence intensity in untreated TYRO3-stained control cells and cells treated with Meleagrin 3 µM (**A**), Meleagrin 5 µM (**B**), Cucurbitacin B 10 µM (**C**), and Cucurbitacin B 20 µM (**D**). Fluorescence intensity is presented as normalized cell count (%) versus FITC-A fluorescence intensity. Treatment with Cucurbitacin B produced more apparent leftward shifts in fluorescence intensity relative to untreated TYRO3-stained cells, particularly at higher concentrations.

The effects of Meleagrin and Cucurbitacin B on TYRO3 expression were evaluated by flow cytometry in HT-29 CRC cells (Figure 2). Representative histograms demonstrated limited changes in TYRO3 fluorescence profiles following Meleagrin treatment at the tested concentrations. Similarly, treatment with 10 µM Cucurbitacin B produced only minor alterations relative to untreated TYRO3-stained cells. In contrast, treatment with 20 µM Cucurbitacin B resulted in a more evident leftward shift in fluorescence intensity, suggesting reduced TYRO3 expression in HT-29 cells. Overall, these findings indicate that modulation of TYRO3 fluorescence was more apparent following treatment with higher concentrations of Cucurbitacin B.

**Figure 3:**
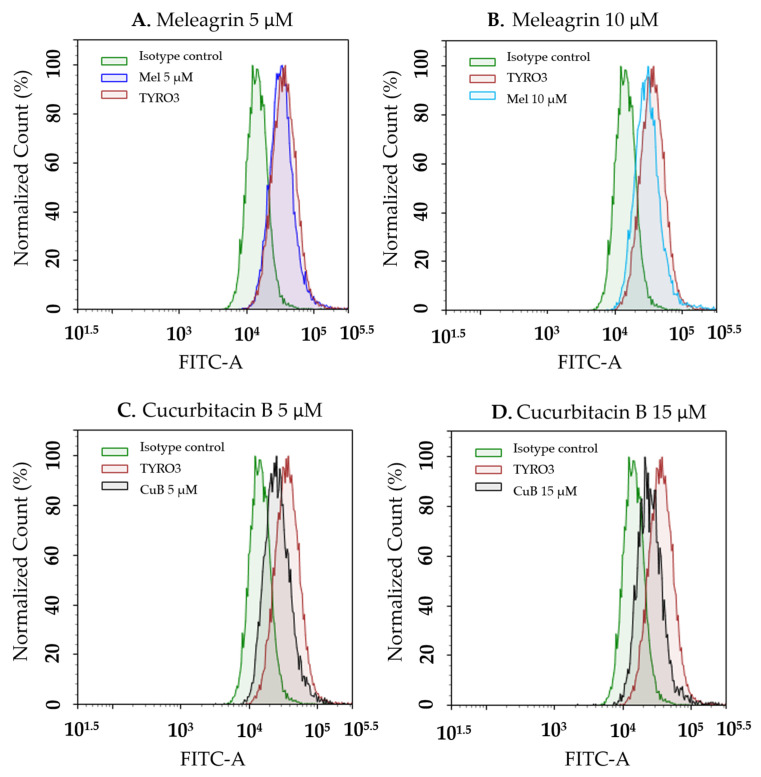
Flow cytometric analysis of TYRO3 fluorescence signal in HCT-116 CRC cells following treatment with Meleagrin and Cucurbitacin B. Representative overlaid histograms showing TYRO3 fluorescence intensity in untreated TYRO3-stained control cells and cells treated with Meleagrin 5 µM (**A**), Meleagrin 10 µM (**B**), Cucurbitacin B 5 µM (**C**), and Cucurbitacin B 15 µM (**D**). Fluorescence intensity is presented as normalized cell count (%) versus FITC-A fluorescence intensity. Treatment with both Meleagrin and Cucurbitacin B induced leftward shifts in fluorescence intensity relative to untreated TYRO3-stained cells, with more apparent changes observed following higher concentrations of Cucurbitacin B.

Flow cytometric analysis was performed to evaluate the effects of Meleagrin and Cucurbitacin B on TYRO3 expression in HCT-116 CRC cells (Figure 3). Representative histograms demonstrated modest leftward shifts in fluorescence intensity following Meleagrin treatment, with more evident changes observed at 10 µM. Similarly, treatment with 5 µM Cucurbitacin B produced only minor alterations relative to untreated TYRO3-stained cells. In contrast, treatment with 15 µM Cucurbitacin B resulted in a clearer leftward shift in fluorescence intensity, suggesting reduced TYRO3 expression at higher concentrations. Overall, these findings indicate that modulation of TYRO3 fluorescence was more apparent in HCT-116 cells following treatment with higher concentrations of Cucurbitacin B.

**Figure 4:**
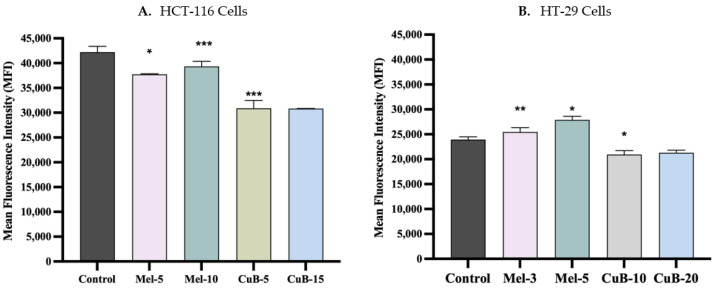
Quantitative analysis of TYRO3 fluorescence intensity in CRC cells following treatment with Meleagrin and Cucurbitacin B. Mean fluorescence intensity (MFI) values of TYRO3-associated fluorescence were determined by flow cytometry in HCT-116 (**A**) and HT-29 (**B**) CRC cells following treatment with Meleagrin or Cucurbitacin B at the indicated concentrations. Data are presented as mean ± SD. Statistical significance was evaluated relative to untreated control cells (* *p* < 0.05, ** *p* < 0.01, *** *p* < 0.001).

Flow cytometric quantification of TYRO3 fluorescence was performed in HCT-116 and HT-29 CRC cells following treatment with Meleagrin and Cucurbitacin B (Figure 4). In HCT-116 cells, Meleagrin treatment produced modest alterations in mean fluorescence intensity (MFI), with a statistically significant reduction observed at 5 µM (* *p* < 0.05), whereas no clear concentration-dependent effect was detected between the tested doses. In contrast, Cucurbitacin B treatment significantly reduced TYRO3 fluorescence at both tested concentrations (* *p* < 0.001), indicating a stronger suppressive effect on TYRO3 expression in HCT-116 cells.

In HT-29 cells, Meleagrin treatment resulted in significant increases in TYRO3-associated fluorescence intensity at both tested concentrations, with a more pronounced effect observed at 3 µM (** *p* < 0.01) and a moderate increase at 5 µM (*p* < 0.05). Conversely, treatment with Cucurbitacin B significantly reduced TYRO3 fluorescence at 10 µM (*p* < 0.05), whereas the reduction observed at 20 µM did not reach statistical significance. Overall, these findings suggest that Cucurbitacin B exerted a more consistent suppressive effect on TYRO3 fluorescence in both CRC cell lines, whereas the response to Meleagrin appeared to be cell line dependent.

#### 2.2.2. Real-Time Quantitative Polymerase Chain Reaction (RT-qPCR) Analysis of TYRO3 Expression

**Figure 5:**
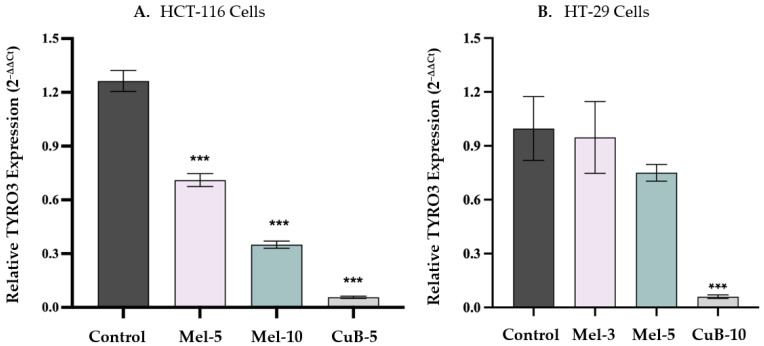
Effects of Meleagrin and Cucurbitacin B on TYRO3 mRNA expression in CRC cells determined by RT-qPCR analysis. Relative TYRO3 mRNA expression levels were evaluated in HCT-116 (**A**) and HT-29 (**B**) CRC cells following treatment with Meleagrin and Cucurbitacin B. Cells were treated with Meleagrin (3, 5, and 10 µM) or Cucurbitacin B (5 and 10 µM) for 24 h prior to RNA isolation. Gene expression levels were normalized to *ACTB* and calculated using the 2^−ΔΔCt^ method relative to untreated control cells. Data are presented as mean ± SD. Statistical significance: *** *p* < 0.001 versus untreated control cells.

The effects of Meleagrin and Cucurbitacin B on TYRO3 gene expression were evaluated by RT-qPCR in HCT-116 and HT-29 CRC cells (Figure 5). In HCT-116 cells, both compounds significantly reduced TYRO3 messenger RNA (mRNA) expression levels compared with untreated controls. The most pronounced reduction was observed following Cucurbitacin B exposure, while Meleagrin also induced significant downregulation at the tested concentrations (*** *p* < 0.001).

In HT-29 cells, TYRO3 mRNA expression was less responsive to treatment than in HCT-116 cells. Although Cucurbitacin B induced a marked reduction in TYRO3 expression (*** *p* < 0.001), Meleagrin produced only modest and non-significant changes. Overall, these findings indicate that HCT-116 cells exhibited a broader transcriptional response to both compounds, whereas in HT-29 cells substantial TYRO3 suppression was primarily observed following Cucurbitacin B treatment.

### 2.3. Effects on Cell Viability and Proliferation

#### 2.3.1. MTT Assay

**Figure 6:**
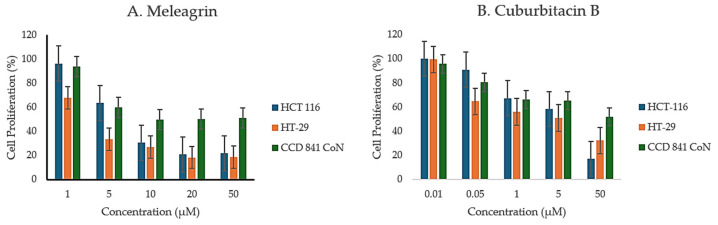
Antiproliferative effects of Meleagrin and Cucurbitacin B in CRC and normal colon epithelial cells determined by MTT assay. HT-29, HCT-116, and CCD 841 CoN cells were treated with increasing concentrations of Meleagrin (**A**) or Cucurbitacin B (**B**) for 48 h, and cell proliferation was evaluated using the MTT assay. Results are expressed as relative cell proliferation (%) compared with untreated control cells.

The antiproliferative effects of Meleagrin and Cucurbitacin B were evaluated in HT-29, HCT-116, and CCD 841 CoN cells using the MTT assay (Figure 6). Both compounds reduced cell proliferation in a concentration-dependent manner, although the magnitude of the response differed among cell lines. Meleagrin produced more pronounced antiproliferative effects in HT-29 cells than in HCT-116 cells, with calculated half maximal inhibitory concentration (IC_50_) values of 3.54 and 6.96 µM, respectively. In contrast, CCD 841 CoN cells exhibited substantially lower sensitivity to Meleagrin (IC_50_ = 49.73 µM). Cucurbitacin B also reduced cell proliferation in all tested cell lines, with HCT-116 cells displaying greater sensitivity (IC_50_ = 5.83 µM) than HT-29 cells (IC_50_ = 16.79 µM). Although CCD 841 CoN cells were affected by Cucurbitacin B, the corresponding IC_50_ value (49.42 µM) remained markedly higher than those observed in CRC cells. Collectively, these findings indicate that both compounds preferentially inhibited CRC cell proliferation relative to normal colon epithelial cells, with Meleagrin exhibiting the largest selectivity window under the tested conditions. Detailed IC_50_ values are provided in Supplementary Table S1.

#### 2.3.2. Real-Time Cell Analysis (xCELLigence)

**Figure 7:**
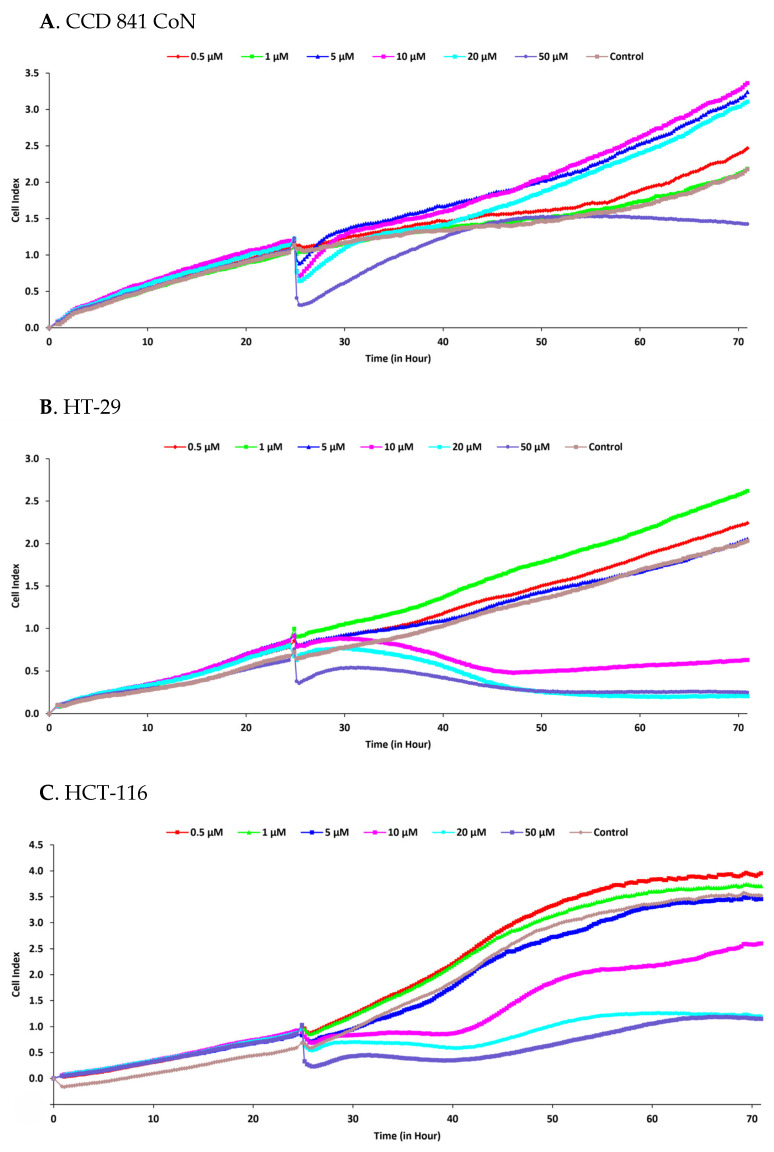
Real-time analysis of the antiproliferative effects of Meleagrin in CRC and normal colon epithelial cells using the xCELLigence system. Cell proliferation dynamics were continuously monitored using the xCELLigence real-time cell analysis system following treatment with different concentrations of Meleagrin. Representative cell index curves of CCD 841 CoN (**A**), HT-29 (**B**), and HCT-116 (**C**) cells are shown over a 72 h monitoring period. Meleagrin produced concentration-dependent alterations in proliferation profiles, with less pronounced inhibitory effects observed in CCD 841 CoN cells at lower concentrations.

The antiproliferative effects of Meleagrin were further evaluated using the xCELLigence real-time cell analysis system in CCD 841 CoN, HT-29, and HCT-116 cells (Figure 7). Dynamic changes in cell index values were continuously monitored for up to 72 h following treatment.

In CCD 841 CoN cells, lower and intermediate concentrations of Meleagrin produced modest inhibitory effects, whereas treatment with 50 µM reduced cell index values during the later stages of the experiment. In HT-29 cells, Meleagrin induced more evident concentration-dependent antiproliferative effects, with marked suppression of proliferation observed at concentrations of 10 µM and above.

Similarly, HCT-116 cells maintained proliferation at lower concentrations of Meleagrin, while higher concentrations produced more apparent reductions in cell index values over time. Overall, real-time proliferation analysis demonstrated that Meleagrin suppresses CRC cell proliferation in a concentration-dependent manner, whereas more limited inhibitory effects were observed in normal colon epithelial cells at lower concentrations.

**Figure 8:**
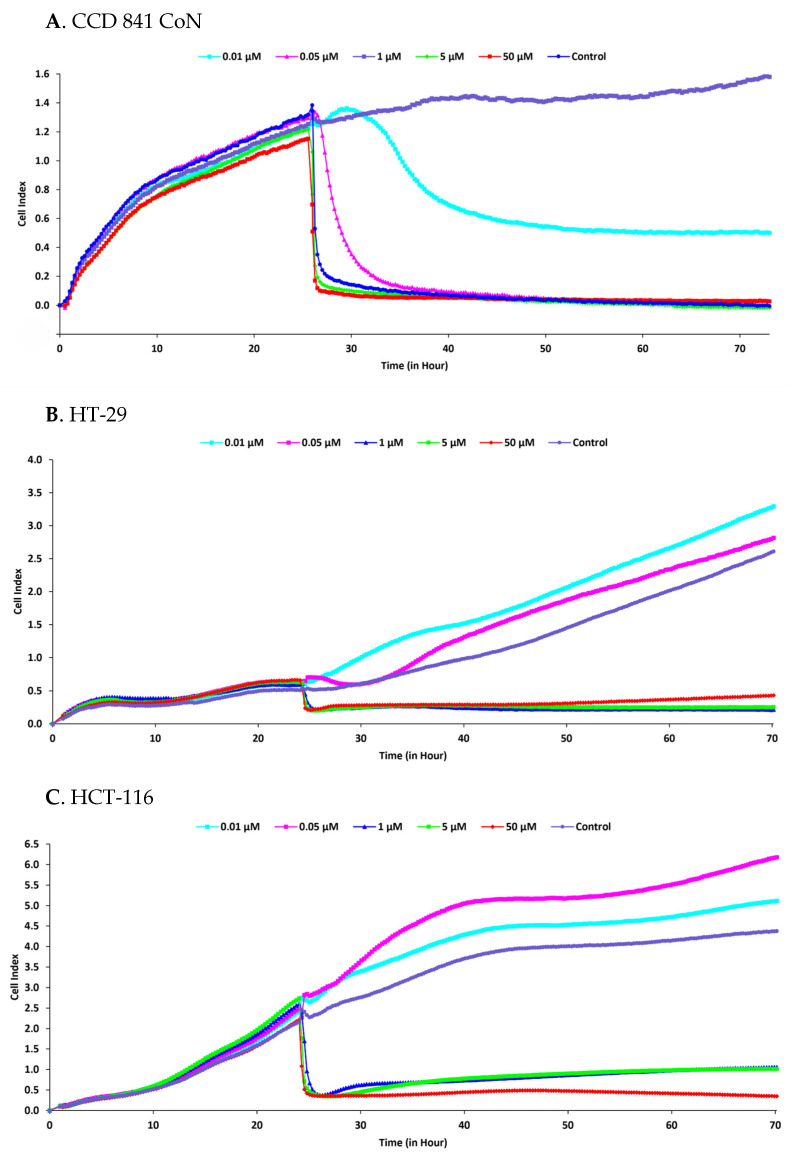
Real-time analysis of the antiproliferative effects of Cucurbitacin B in CRC and normal colon epithelial cells using the xCELLigence system. Cell proliferation dynamics were continuously monitored using the xCELLigence real-time cell analysis system following treatment with different concentrations of Cucurbitacin B. Representative cell index curves of CCD 841 CoN (**A**), HT-29 (**B**), and HCT-116 (**C**) cells are shown over a 72 h monitoring period. Lower concentrations of Cucurbitacin B produced relatively different effects on proliferation dynamics, whereas concentrations of 1 µM and above markedly suppressed cell proliferation across the tested cell lines.

The antiproliferative effects of Cucurbitacin B were further investigated using the xCELLigence real-time cell analysis system in CCD 841 CoN, HT-29, and HCT-116 cells (Figure 8). Dynamic changes in cell index values were continuously monitored for up to 72 h following treatment.

In CCD 841 CoN cells, lower concentrations of Cucurbitacin B (0.01 and 0.05 µM) maintained proliferative activity and exceeded untreated control levels during later time points, whereas concentrations of 1 µM and above induced a rapid and sustained decline in cell index values following treatment. In HT-29 cells, lower concentrations similarly supported continued proliferation, while concentrations of 1 µM and higher markedly suppressed cell growth throughout the monitoring period.

Likewise, HCT-116 cells exhibited enhanced proliferation at lower concentrations of Cucurbitacin B compared with untreated controls, whereas higher concentrations produced substantial reductions in cell index values over time. Overall, real-time proliferation analysis demonstrated that Cucurbitacin B exerts concentration-dependent antiproliferative effects at higher concentrations, while lower concentrations produced distinct effects on proliferation dynamics across the tested cell lines.

### 2.4. Induction of Apoptosis

#### 2.4.1. Annexin V-Fluorescein Isothiocyanate (FITC)/Propidium Iodide (PI) Analysis

**Figure 9:**
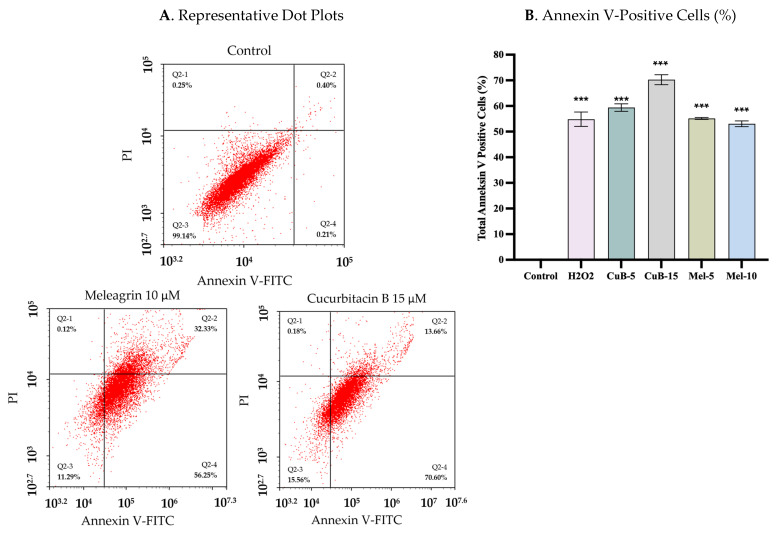
Annexin V-FITC/PI analysis of apoptotic cell death in HCT-116 CRC cells following treatment with Meleagrin and Cucurbitacin B. Representative flow cytometry dot plots of untreated control cells and HCT-116 cells treated with Meleagrin 10 µM or Cucurbitacin B 15 µM are shown (**A**). Quantitative analysis of Annexin V-positive cell populations is presented in panel (**B**). H_2_O_2_ was used as a positive control. Data are presented as mean ± SD. Statistical significance: *** *p* < 0.001 versus untreated control cells.

Apoptotic cell death was evaluated by Annexin V-FITC/PI staining in HCT-116 CRC cells following treatment with Meleagrin and Cucurbitacin B (Figure 9). Representative flow cytometry dot plots demonstrated increased Annexin V-positive cell populations in treated groups compared with untreated controls. Among the tested conditions, Cucurbitacin B at 15 µM produced the most pronounced apoptotic response, whereas Meleagrin 10 µM induced more moderate alterations in Annexin V-positive cell populations.

Quantitative analysis demonstrated significant increases in Annexin V-positive cells following treatment with both compounds relative to untreated controls. The highest apoptotic response was observed in cells treated with Cucurbitacin B 15 µM (*** *p* < 0.001). Overall, these findings indicate that Cucurbitacin B induced a stronger apoptotic response than Meleagrin in HCT-116 cells.

**Figure 10:**
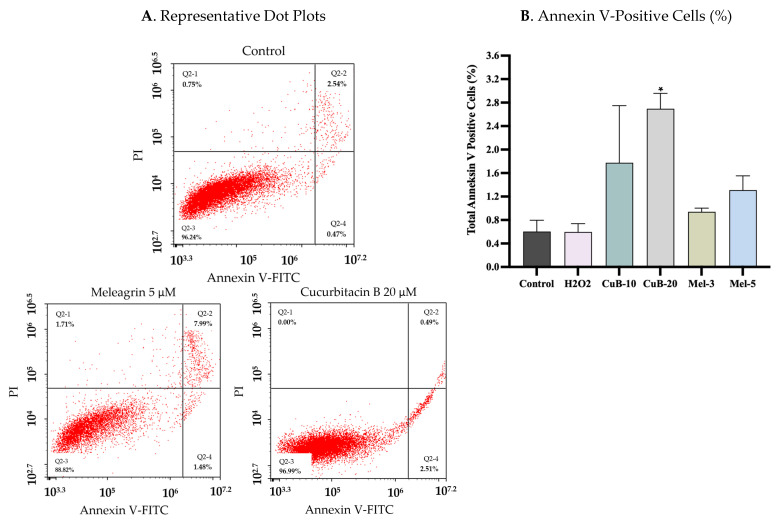
Annexin V-FITC/PI analysis of apoptotic cell death in HT-29 CRC cells following treatment with Meleagrin and Cucurbitacin B. Representative flow cytometry dot plots of untreated control cells and HT-29 cells treated with Meleagrin 5 µM or Cucurbitacin B 20 µM are shown (**A**). Quantitative analysis of total Annexin V-positive cell populations is presented in panel (**B**). H_2_O_2_ was used as a positive control. Data are presented as mean ± SD. Statistical significance: * *p* < 0.05 versus untreated control cells.

Annexin V-FITC/PI flow cytometric analysis was performed to evaluate apoptotic cell populations in HT-29 CRC cells following treatment with Meleagrin and Cucurbitacin B (Figure 10). Representative dot plots demonstrated limited alterations in Annexin V-positive cell populations following Meleagrin treatment relative to untreated controls. In contrast, treatment with Cucurbitacin B 20 µM produced more apparent increases in Annexin V-positive cells, although the overall apoptotic response remained lower than that observed in HCT-116 cells.

Quantitative analysis demonstrated a statistically significant increase in Annexin V-positive cell populations following treatment with Cucurbitacin B (* *p* < 0.05), whereas Meleagrin treatment produced only modest alterations in apoptotic cell populations. Overall, these findings suggest that HT-29 cells exhibited a weaker apoptotic response to the tested compounds than HCT-116 cells under the same experimental conditions.

#### 2.4.2. Caspase-3/7 Activity

**Figure 11:**
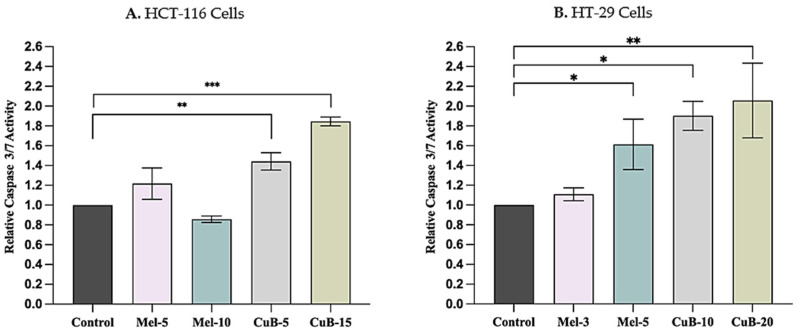
Effects of Meleagrin and Cucurbitacin B on caspase-3/7 activity in CRC cells. Relative caspase-3/7 activity was evaluated in HCT-116 (**A**) and HT-29 (**B**) CRC cells following treatment with Meleagrin and Cucurbitacin B. Caspase-3/7 activity is expressed relative to untreated control cells. Data are presented as mean ± SD. Statistical significance was evaluated relative to untreated control cells (* *p* < 0.05, ** *p* < 0.01, *** *p* < 0.001).

Caspase-3/7 activity was evaluated in HCT-116 and HT-29 CRC cells following treatment with Meleagrin and Cucurbitacin B (Figure 11). In HCT-116 cells, Meleagrin produced limited alterations in caspase-3/7 activity, whereas Cucurbitacin B treatment significantly increased caspase-3/7 activity relative to untreated controls. The highest increase in caspase-3/7 activity was observed following treatment with Cucurbitacin B 15 µM (*** *p* < 0.001).

In HT-29 cells, Meleagrin 5 µM significantly increased caspase-3/7 activity compared with untreated controls (* *p* < 0.05), while lower concentrations produced limited effects. Cucurbitacin B treatment also elevated caspase-3/7 activity in HT-29 cells, with the greatest increase observed at 20 µM (** *p* < 0.01). Overall, these findings indicate that Cucurbitacin B produced greater increases in caspase-3/7 activity than Meleagrin.

#### 2.4.3. Mitochondrial Membrane Potential (JC-1 Assay)

**Figure 12:**
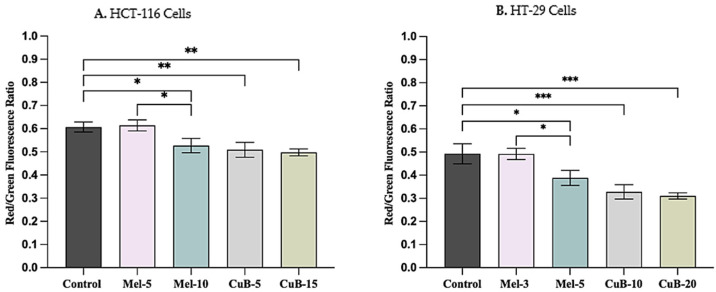
Effects of Meleagrin and Cucurbitacin B on mitochondrial membrane potential in CRC cells determined by JC-1 assay. Mitochondrial membrane potential (ΔΨm) was evaluated in HCT-116 (**A**) and HT-29 (**B**) CRC cells following treatment with Meleagrin and Cucurbitacin B. Results are expressed as the red/green fluorescence ratio relative to untreated control cells. Data are presented as mean ± SD. Statistical significance was evaluated relative to untreated control cells (* *p* < 0.05, ** *p* < 0.01, *** *p* < 0.001).

Changes in mitochondrial membrane potential (ΔΨm) were evaluated using the JC-1 assay in HCT-116 and HT-29 CRC cells following treatment with Meleagrin and Cucurbitacin B (Figure 12). In HCT-116 cells, treatment with Meleagrin 10 µM significantly reduced the red/green fluorescence ratio compared with untreated controls (* *p* < 0.05), whereas Meleagrin 5 µM produced limited changes. Similarly, both tested concentrations of Cucurbitacin B significantly reduced the red/green fluorescence ratio (** *p* < 0.01).

In HT-29 cells, treatment with Meleagrin 5 µM significantly decreased the red/green fluorescence ratio relative to untreated controls (* *p* < 0.05), whereas lower concentrations produced modest effects. Cucurbitacin B treatment also reduced the red/green fluorescence ratio in HT-29 cells, with the strongest reduction observed following treatment with Cucurbitacin B 20 µM (*** *p* < 0.001). Overall, these findings indicate that Cucurbitacin B produced greater reductions in mitochondrial membrane potential than Meleagrin.

### 2.5. Effects on Cell Migration

**Figure 13:**
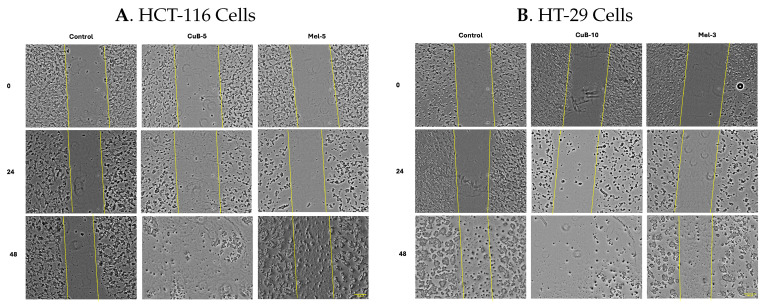
Effects of Meleagrin and Cucurbitacin B on wound closure dynamics in CRC cells determined by wound healing assay. Representative wound healing assay images obtained from HCT-116 (**A**) and HT-29 (**B**) CRC cells following treatment with Meleagrin or Cucurbitacin B. Images were captured at 0, 24, and 48 h after treatment. Yellow lines indicate wound boundaries at each time point. Higher concentrations of Cucurbitacin B were associated with reduced wound closure and decreased cell density at later time points. Scale bar: 100 µm.

Cell migration was evaluated using a wound healing assay in HCT-116 and HT-29 CRC cells following treatment with Meleagrin and Cucurbitacin B (Figure 13). In untreated control groups, progressive wound closure was observed over time, indicating preserved migratory activity in both cell lines.

In HCT-116 cells, Meleagrin treatment produced small alterations in migratory behavior relative to untreated controls. In contrast, treatment with Cucurbitacin B 15 µM resulted in more apparent inhibition of wound closure, particularly at 48 h, where wider remaining wound areas were observed. In addition, higher concentrations of Cucurbitacin B were associated with reduced cell density and partial disruption of monolayer integrity at later time points.

Similarly, HT-29 cells treated with Meleagrin exhibited weaker alterations in wound closure over time. Treatment with Cucurbitacin B 20 µM produced clearer suppression of wound closure and was accompanied by marked reductions in cell density at 48 h. Overall, these findings suggest that Cucurbitacin B exerted greater effects on wound healing capacity than Meleagrin.

**Figure 14:**
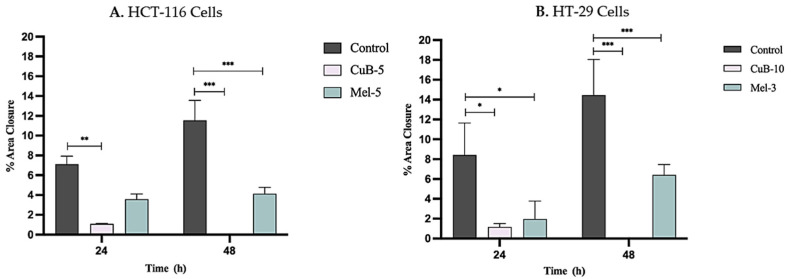
Quantitative analysis of wound closure in CRC cells following treatment with Meleagrin and Cucurbitacin B. Wound closure (%) was quantified at 24 and 48 h in HCT-116 (**A**) and HT-29 (**B**) CRC cells following treatment with Meleagrin or Cucurbitacin B at the indicated concentrations. Data are presented as mean ± SD. Statistical significance was evaluated between groups (* *p* < 0.05, ** *p* < 0.01, *** *p* < 0.001).

The effects of Meleagrin and Cucurbitacin B on CRC cell migration were quantitatively evaluated using a wound healing assay in HCT-116 and HT-29 cells (Figure 14). Wound closure progressively increased over time in untreated control groups, indicating preserved migratory activity in both cell lines.

In HCT-116 cells, treatment with Cucurbitacin B (5 µM) significantly reduced wound closure at both 24 h (** *p* < 0.01) and 48 h (*** *p* < 0.001) compared with untreated controls, indicating marked inhibition of migratory activity. Meleagrin (5 µM) also reduced wound closure relative to controls, although the effect was less pronounced than that observed with Cucurbitacin B. At 48 h, wound closure remained significantly lower in Cucurbitacin B-treated cells than in both control and Meleagrin-treated groups (* *p* < 0.001).

Similarly, in HT-29 cells, Cucurbitacin B (10 µM) significantly suppressed wound closure at both 24 h (* *p* < 0.05) and 48 h (* *p* < 0.001) relative to untreated controls. Meleagrin (3 µM) produced a moderate reduction in wound closure compared with controls; however, its inhibitory effect remained weaker than that of Cucurbitacin B. Overall, these findings indicate that Cucurbitacin B exerted stronger inhibitory effects on migratory behavior than Meleagrin in both CRC cell lines.

### 2.6. Molecular Docking and Absorption, Distribution, Metabolism, and Excretion (ADME) Analysis

**Figure 15:**
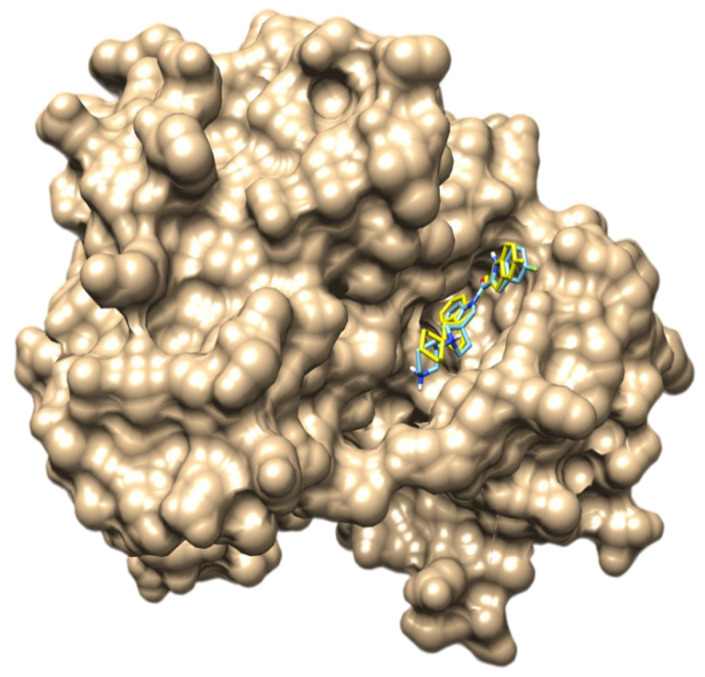
Redocking validation of the native inhibitor within the TYRO3 kinase domain (PDB ID: 3QUP). Surface representation of the TYRO3 kinase domain showing the superposition of the crystallographic ligand and the redocked pose. The low RMSD value (1.240 Å) demonstrated successful reproduction of the native binding mode and validated the reliability of the applied docking protocol.

**Figure 16:**
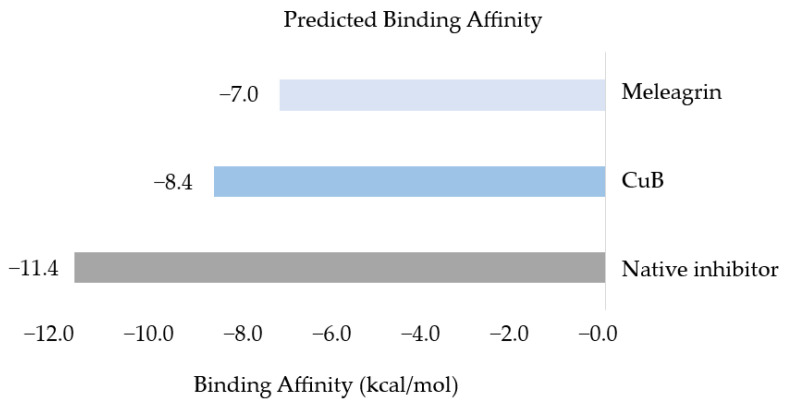
Predicted binding affinities of the investigated compounds against the TYRO3 kinase domain. Binding affinity values obtained from molecular docking analyses of the native inhibitor, Cucurbitacin B (CuB), and Meleagrin against the TYRO3 kinase domain (PDB ID: 3QUP). Cucurbitacin B exhibited a more favorable predicted binding affinity than Meleagrin.

To support the experimental findings, an initial virtual screening workflow using iGEMDOCK was performed to identify candidate TYRO3-modulating compounds from the investigated natural compound library. Based on preliminary screening results and experimental accessibility, Cucurbitacin B and Meleagrin were selected for further computational and biological evaluation. Refined molecular docking analyses were subsequently performed against the TYRO3 kinase domain (PDB ID: 3QUP). Redocking of the co-crystallized inhibitor successfully reproduced the native binding pose with an RMSD value of 1.240 Å, validating the reliability of the applied docking protocol (Figure 15) [43,44,45]. Among the investigated compounds, Cucurbitacin B exhibited a more favorable predicted binding affinity (−8.4 kcal/mol) than Meleagrin (−7.0 kcal/mol), although both compounds demonstrated lower docking scores than the native inhibitor (−11.4 kcal/mol) (Figure 16).

**Figure 17:**
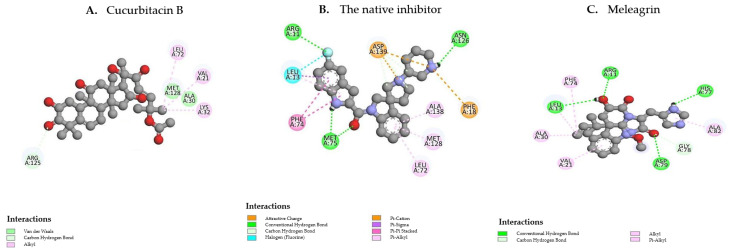
Protein–ligand interaction profiles within the TYRO3 kinase domain. Two-dimensional interaction maps show the predicted interactions of Cucurbitacin B (**A**), the native inhibitor (**B**), and Meleagrin (**C**) within the TYRO3 catalytic pocket. Cucurbitacin B established predominantly hydrophobic interactions involving residues VAL21, LYS32, and LEU72, whereas the native inhibitor formed additional electrostatic and aromatic interactions. Meleagrin formed ten interactions including four conventional hydrogen bonds with ARG11, ASP79, HIS77, and LEU13, and hydrophobic contacts with PHE74, VAL21, ALA30, LEU13, and ALA82.

Protein–ligand interaction analyses revealed that Cucurbitacin B established predominantly hydrophobic interactions within the TYRO3 catalytic pocket, particularly involving residues VAL21, LYS32, and LEU72. In contrast, the native inhibitor formed additional electrostatic and aromatic interactions, suggesting a more optimized active-site occupation profile. Nevertheless, the shared interaction with LEU72 may indicate a conserved hydrophobic hotspot relevant for ligand accommodation within the TYRO3 kinase domain (Figure 17) [46,47].

Meleagrin established ten interactions within the TYRO3 binding site. Four conventional hydrogen bonds were identified: ARG11 and ASP79 acted as hydrogen bond donors toward the O4 and O2 acceptor atoms of the ligand (2.148 Å and 2.159 Å, respectively), while the ligand contributed as a donor via H15 and H23 atoms, forming contacts with HIS77 (2.532 Å) and LEU13 (2.867 Å). A carbon–hydrogen bond between GLY78 and Meleagrin O2 (2.126 Å) provided supplementary polar stabilization. Hydrophobic contacts included alkyl interactions with ALA30 and LEU13, and pi–alkyl interactions with PHE74, VAL21, and ALA82 (Figure 17).

**Figure 18:**
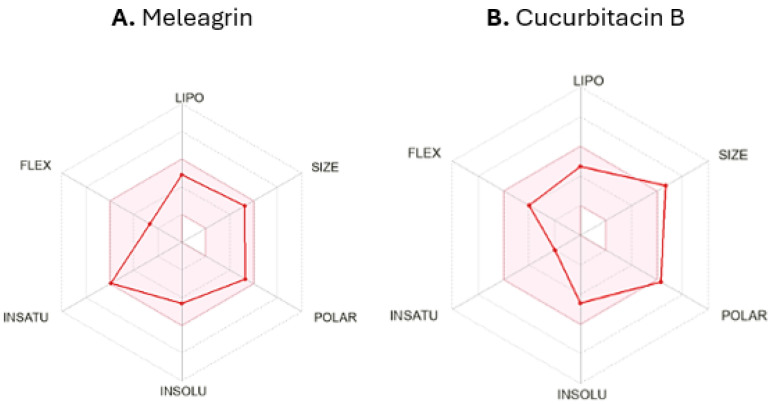
SwissADME bioavailability radar plots of Meleagrin and Cucurbitacin B. Bioavailability radar plots illustrating the predicted physicochemical and drug-likeness properties of Meleagrin (**A**) and Cucurbitacin B (**B**), including lipophilicity (LIPO), molecular size (SIZE), polarity (POLAR), solubility (INSOLU), flexibility (FLEX), and saturation (INSATU). The pink shaded area represents the optimal physicochemical space associated with oral bioavailability.

SwissADME analyses indicated that Meleagrin exhibited a more favorable predicted drug-likeness and oral bioavailability profile, including high gastrointestinal absorption and compliance with all evaluated drug-likeness filters. In contrast, Cucurbitacin B demonstrated lower predicted gastrointestinal absorption and one Lipinski violation related to molecular weight, along with additional Ghose and Egan filter violations. Both compounds were predicted to be P-glycoprotein substrates and triggered two Brenk structural alerts each, including Michael acceptor moieties. Detailed physicochemical, pharmacokinetic, and drug-likeness parameters are provided in Supplementary Table S3. These findings suggest that further pharmacokinetic and toxicological investigations will be required to evaluate the translational potential of both compounds [48]. The predicted physicochemical and drug-likeness properties are further illustrated by the SwissADME bioavailability radar plots shown in Figure 18.

### 2.7. Molecular Dynamics Simulation of TYRO3–Ligand Complexes

**Figure 19:**
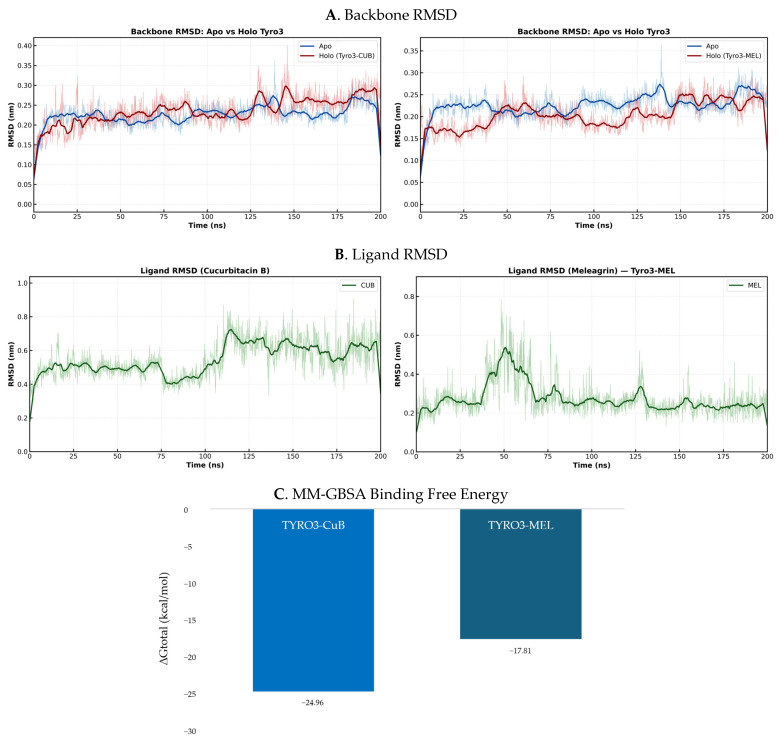
Molecular dynamics simulation analyses of TYRO3 complexes with Cucurbitacin B and Meleagrin. (**A**) Backbone RMSD profiles of apo TYRO3 and holo TYRO3–ligand complexes during 200 ns molecular dynamics simulations. TYRO3–Cucurbitacin B (**left**) and TYRO3–Meleagrin (**right**) complexes remained structurally stable throughout the simulation period. (**B**) Ligand RMSD profiles of Cucurbitacin B (**left**) and Meleagrin (**right**) within the TYRO3 binding pocket over 200 ns. Both ligands remained associated with the binding site during the simulation, with Meleagrin exhibiting lower RMSD fluctuations and a more stable binding orientation than Cucurbitacin B.

To further validate the docking results and assess the dynamic stability of ligand binding, 200 ns molecular dynamics (MD) simulations were performed for TYRO3 complexes with Cucurbitacin B and Meleagrin (Figure 19). Backbone RMSD analyses demonstrated that both complexes remained stable throughout the simulation period, with average RMSD values of 0.236 ± 0.037 nm for TYRO3–Cucurbitacin B and 0.205 ± 0.032 nm for TYRO3–Meleagrin (Figure 19). These findings indicate that both TYRO3–ligand complexes remained structurally stable throughout the simulation period and that ligand binding did not induce major conformational destabilization of the protein.

Ligand RMSD analyses further demonstrated stable binding behavior for both compounds (Figure 19). Cucurbitacin B exhibited an average ligand RMSD of 0.547 ± 0.101 nm, whereas Meleagrin displayed a lower average ligand RMSD of 0.276 ± 0.091 nm, indicating a particularly stable binding orientation throughout the simulation period. Both ligands remained associated with the binding pocket throughout the simulation, supporting the stability of the docked binding poses.

To estimate binding strength, molecular mechanics generalized Born surface area (MM-GBSA) calculations were performed using the final 100 ns of the MD trajectories (Figure 19). Cucurbitacin B exhibited a more favorable binding free energy (ΔGtotal = −24.96 ± 0.50 kcal/mol) than Meleagrin (ΔGtotal = −17.81 ± 0.22 kcal/mol). Nevertheless, both compounds demonstrated negative ΔG values consistent with stable and energetically favorable interactions with TYRO3. Collectively, the molecular dynamics and MM-GBSA analyses corroborate the docking results and support the ability of both compounds to establish stable interactions within the TYRO3 kinase domain. Additional RMSF, radius of gyration (Rg), solvent-accessible surface area (SASA), protein–ligand contact, center-of-mass distance, hydrogen-bond occupancy, and residue-wise MM-GBSA decomposition analyses are provided in Supplementary Figure S2. A summary of the molecular dynamics simulation parameters and binding stability metrics is provided in Supplementary Table S4.

## 3. Discussion

The present study demonstrates that Cucurbitacin B and Meleagrin exert multifaceted anticancer effects in CRC cells, including suppression of proliferation and migratory activity together with induction of apoptotic responses, accompanied by modulation of TYRO3 expression at both the mRNA and protein levels. Although accumulating evidence has identified TYRO3 as a promising therapeutic target in CRC, studies investigating naturally derived compounds capable of modulating TYRO3-associated signaling remain limited [19,26,49]. Collectively, the present findings therefore provide novel evidence demonstrating an association between the anticancer effects of Cucurbitacin B and Meleagrin and modulation of TYRO3 expression in CRC cells and provide a rationale for further investigation of naturally derived TYRO3-modulating compounds in CRC.

Accumulating evidence identifies TYRO3 as a functionally relevant regulator of CRC progression and therapeutic resistance [19,22,25]. Consistent with our flow cytometric findings, previous studies evaluating TYRO3 expression across different CRC cell lines similarly reported relatively high TYRO3 expression in HCT-116 and LOVO cells, whereas HT-29 cells exhibited comparatively lower basal TYRO3 levels [22]. In the present study, HCT-116 cells likewise demonstrated relatively higher basal TYRO3-associated fluorescence intensity than HT-29 cells, supporting the presence of cell line-dependent variability in TYRO3 expression among CRC models. Previous reports similarly suggested that heterogeneous TYRO3 expression patterns among CRC subtypes may contribute to variability in oncogenic signaling dependency, treatment sensitivity, and drug resistance [20,21,22].

Although both compounds affected TYRO3 expression, Cucurbitacin B consistently produced more evident modulation together with stronger antiproliferative and pro-apoptotic effects than Meleagrin under the tested conditions. These observations are broadly consistent with the reported biological consequences of TYRO3 suppression in CRC models [19,23,26]. In this context, the present findings suggest that TYRO3 modulation may be associated, at least in part, with the observed biological effects of these compounds in CRC cells. The differential effects observed between Cucurbitacin B and Meleagrin further indicate that the extent of TYRO3 modulation may depend not only on basal receptor expression but also on compound-specific biological properties and their potential to influence TYRO3-associated cellular processes.

Flow cytometric analyses demonstrated relatively limited alterations in TYRO3-associated fluorescence profiles compared with the more pronounced reductions observed at the mRNA level, particularly following Cucurbitacin B treatment. This discrepancy may reflect differences between transcriptional regulation and surface protein dynamics, including post-transcriptional regulation, receptor turnover kinetics, and the fact that flow cytometry measures surface-associated TYRO3 rather than total cellular TYRO3 protein. Delayed changes in receptor abundance following transcriptional suppression may also contribute to the observed differences. Nevertheless, relatively greater reductions in TYRO3-associated fluorescence were observed following exposure to higher concentrations of Cucurbitacin B, particularly in HCT-116 cells, whereas Meleagrin produced only modest alterations under the tested conditions. Interestingly, Meleagrin increased TYRO3-associated fluorescence in HT-29 cells despite its antiproliferative activity. This observation may reflect altered receptor trafficking, compensatory surface accumulation, changes in antibody accessibility, or other post-transcriptional regulatory mechanisms rather than an increase in TYRO3 signaling activity. Because receptor phosphorylation and downstream signaling were not directly assessed, increased surface-associated fluorescence should not be interpreted as evidence of enhanced TYRO3 function. Several studies have similarly highlighted that TYRO3-associated oncogenic phenotypes may be influenced not only by receptor expression levels but also by receptor activation status and intracellular signaling activity [19,22,26]. Because TYRO3 protein abundance was not evaluated by Western blotting, and flow cytometry primarily measures cell-surface-associated TYRO3 rather than total cellular TYRO3 protein, the discrepancy between mRNA expression and flow cytometric measurements should be interpreted cautiously, and the present findings should not be interpreted as representing total cellular TYRO3 protein abundance. Future studies assessing total protein levels, receptor phosphorylation, subcellular localization, and downstream signaling will be necessary to clarify whether these findings reflect altered TYRO3 abundance, localization, or receptor trafficking dynamics. In this context, the present findings suggest that even relatively modest changes in TYRO3 protein expression may be associated with measurable antiproliferative and pro-apoptotic effects in CRC cells.

In silico analyses further complemented the experimental findings by demonstrating plausible interactions of both compounds within the TYRO3 kinase pocket. Notably, Cucurbitacin B exhibited a more favorable predicted binding profile than Meleagrin, which is consistent with its stronger effects on TYRO3 modulation and the associated antiproliferative and pro-apoptotic responses observed in CRC cells. Despite its lower docking score, Meleagrin formed a qualitatively broader set of specific polar contacts within the TYRO3 binding site, including four conventional hydrogen bonds with ARG11, ASP79, HIS77, and LEU13, which were absent from the Cucurbitacin B interaction profile. This suggests a degree of directional specificity that binding energy alone does not fully capture. Previous structural modelling and docking studies have demonstrated that specific ligand–TYRO3 interactions play an important role in receptor activation and biological function [50,51]. In this context, the present findings raise the possibility that differences in the predicted interaction profiles of Cucurbitacin B and Meleagrin may contribute to their distinct biological activities. However, additional biochemical and biophysical studies are required to confirm direct target engagement and characterize the precise nature of these interactions. Although these computational analyses support the plausibility of TYRO3 engagement, they primarily served to complement the experimental observations and to generate hypotheses regarding potential molecular interactions, rather than providing direct evidence of target engagement.

Supplementary SwissADME analyses further suggested differences in the predicted pharmacokinetic profiles of the compounds investigated. Meleagrin exhibited high predicted gastrointestinal absorption and full compliance with the evaluated drug-likeness filters, whereas Cucurbitacin B showed lower predicted gastrointestinal absorption and several drug-likeness violations, largely related to its higher molecular weight and physicochemical complexity (Table S2). Both compounds were predicted to be P-glycoprotein substrates and triggered two Brenk structural alerts, including Michael acceptor moieties. Although these predictions require experimental validation, they suggest that the superior biological activity observed for Cucurbitacin B may not necessarily be accompanied by equally favorable pharmacokinetic properties. Conversely, the more favorable predicted drug-likeness profile of Meleagrin may warrant further investigation despite its comparatively weaker biological activity [48]. This observation highlights the importance of considering both biological potency and developability-related properties during early-stage candidate evaluation.

The molecular dynamics simulations further strengthened the docking findings by demonstrating stable retention of both compounds within the TYRO3 binding pocket over a 200 ns simulation period. Although Cucurbitacin B exhibited a more favorable MM-GBSA binding free energy than Meleagrin, both ligands maintained stable protein–ligand interactions throughout the simulations, supporting the overall stability of the predicted binding modes. Interestingly, Meleagrin displayed the lowest ligand RMSD values among the investigated TYRO3 complexes, suggesting a highly stable binding orientation despite its comparatively weaker predicted binding energy. This observation highlights that binding stability and binding affinity do not necessarily correlate directly and may reflect different aspects of ligand–receptor recognition. Consistent with the docking analyses, Cucurbitacin B demonstrated stronger predicted affinity, whereas Meleagrin formed a broader network of specific polar interactions. Together, these findings suggest that the two compounds may engage TYRO3 through distinct interaction patterns, with Cucurbitacin B favoring stronger energetic stabilization and Meleagrin exhibiting a more conformationally stable binding mode. Such differences may contribute to the distinct biological responses observed experimentally; however, these computational findings remain predictive in nature and do not, by themselves, establish direct target engagement between the investigated compounds and TYRO3.

The antiproliferative effects observed in the present study are generally consistent with previous reports describing the anticancer activities of Cucurbitacin B and Meleagrin in multiple tumor models [34,35,38]. The broad biological activity of Cucurbitacin B has been attributed to its ability to simultaneously affect multiple growth- and survival-associated signaling networks [37,38]. Published studies indicate that Meleagrin acts through kinase-associated mechanisms in multiple cancer models [35,47]. These observations suggest that both compounds are capable of targeting signaling networks involved in tumor cell growth and survival.

In the present study, both compounds reduced CRC cell proliferation; however, the magnitude of the response differed according to both compound and cellular context. HCT-116 cells exhibited greater sensitivity to Cucurbitacin B than HT-29 cells, particularly at lower concentrations. This observation may be related to intrinsic molecular differences between the two CRC models, including differences in basal TYRO3 expression, genetic background, and signaling pathway dependency. Previous studies similarly reported heterogeneous TYRO3 expression patterns among CRC cell lines and demonstrated relatively higher TYRO3 expression in HCT-116 cells compared with HT-29 cells, suggesting that cellular dependence on TYRO3-associated signaling may vary among CRC subtypes [20,21,22]. Such differences may partially explain the stronger biological responses observed in HCT-116 cells throughout the present study.

Real-time proliferation analyses using the xCELLigence system further supported these findings and provided additional insight into the temporal dynamics of compound activity. Higher concentrations of Cucurbitacin B produced rapid and sustained reductions in cell index values in both CRC cell lines, whereas lower concentrations generated more variable proliferation profiles over time. Interestingly, transient maintenance or modest increases in proliferative activity were observed under some low-dose conditions, suggesting that partial pathway modulation may not be sufficient to overcome compensatory survival mechanisms. The stronger antiproliferative activity of Cucurbitacin B is also consistent with its reported ability to simultaneously modulate multiple tumor-promoting signaling pathways [37,38]. In contrast, Meleagrin produced more gradual antiproliferative responses, which may reflect differences in target specificity, intracellular target engagement, or the extent of signaling perturbation induced by the compound. Collectively, these findings suggest that TYRO3 modulation alone is unlikely to fully explain the observed biological effects and that additional compound-specific mechanisms may contribute to the differential responses observed between Cucurbitacin B and Meleagrin.

An additional observation of potential translational relevance was the differential response of normal colon epithelial cells. Although Cucurbitacin B produced the strongest antiproliferative effects in CRC cells, it also markedly reduced the viability of CCD 841 CoN cells in both MTT and real-time proliferation analyses. In contrast, Meleagrin maintained antiproliferative activity in CRC cells while exerting lower effects on normal epithelial cells. These findings suggest a potentially more favorable selectivity profile for Meleagrin under the tested conditions. Nevertheless, because dedicated toxicity analyses were not performed in the present study, additional investigations using normal-cell toxicity assays and in vivo models will be necessary to more accurately define the therapeutic window and translational potential of these compounds.

The apoptotic findings obtained in the present study are generally consistent with previous reports describing the pro-apoptotic activity of Cucurbitacin B in colorectal and other cancer models. Cucurbitacin B has been shown to induce apoptosis through multiple mechanisms, including reactive oxygen species accumulation, mitochondrial dysfunction, caspase activation, and suppression of pro-survival signaling pathways such as STAT3- and PI3K/AKT-associated signaling [38,39]. These multitarget effects are thought to contribute to its potent anticancer activity across a broad range of tumor types.

In agreement with these observations, Cucurbitacin B produced substantially stronger apoptotic responses than Meleagrin in the present study. The greater sensitivity of HCT-116 cells was particularly notable and may be related to the comparatively higher basal TYRO3 expression observed in this cell line together with the more evident modulation of TYRO3 expression following compound exposure. Experimental evidence suggests that TYRO3 signaling promotes cell survival, therapeutic resistance, and protection from apoptosis through activation of multiple survival-associated pathways [20,22,23]. Therefore, CRC cells exhibiting greater dependence on TYRO3 signaling may be expected to display increased apoptotic susceptibility following disruption of this pathway.

Interestingly, the differences observed between HCT-116 and HT-29 cells suggest that apoptotic responsiveness is not solely determined by TYRO3 expression levels but is likely influenced by additional cell line-specific molecular characteristics. Differences in genetic background, signaling network dependency, and intrinsic apoptotic threshold may all contribute to the heterogeneous responses observed between CRC models. Similarly, the relatively limited apoptotic activity of Meleagrin despite its measurable effects on proliferation and TYRO3 expression suggests that suppression of cell growth and induction of apoptosis may not necessarily occur to the same extent and may involve partially distinct biological mechanisms.

Collectively, these findings support the notion that Cucurbitacin B exerts stronger pro-apoptotic effects than Meleagrin in CRC cells and further suggest that modulation of TYRO3-associated survival signaling may represent one component of the biological responses induced by this compound.

The wound healing assay revealed distinct biological profiles for the two compounds. Cucurbitacin B produced a more substantial suppression of wound closure than Meleagrin, which is generally consistent with previous reports describing its ability to interfere with EMT-associated phenotypes, invasive behavior, and metastatic progression in CRC and other cancer models [38,40]. Nevertheless, these findings should be interpreted with caution because wound closure is influenced by both cell migration and proliferation. Although the assay was conducted under serum-free conditions to minimize proliferative activity, the marked antiproliferative effects of Cucurbitacin B and Meleagrin may have partially contributed to the reduced wound closure observed in the treated groups. Accordingly, the present results should not be considered definitive evidence of migration-specific inhibition, but rather as indicating an overall impairment of wound closure resulting from reduced proliferation, altered migratory behavior, or a combination of both processes.

The comparatively modest effects of Meleagrin may therefore reflect a weaker influence on migration-associated processes under the tested conditions. However, this cannot be distinguished from its effects on cell proliferation using the present experimental design. The observed changes are broadly consistent with previous studies implicating TYRO3 signaling in CRC cell motility, invasion, and EMT-associated phenotypes [22,25,26]. However, while reduced wound closure occurred concurrently with alterations in TYRO3 expression, the current findings do not establish that TYRO3 directly mediates this response. Further migration-specific (Transwell migration and Matrigel invasion assays) and TYRO3-targeted functional studies will be required to clarify this relationship.

An important strength of the present study is the integration of multiple complementary experimental approaches, including proliferation assays, flow cytometry, RT-qPCR, apoptosis analyses, wound healing assays, and real-time cell monitoring. The combined evaluation of these parameters enabled a broader characterization of the biological responses associated with TYRO3 modulation in CRC cells and facilitated integrated interpretation of proliferation, migration, apoptosis, and expression-related findings.

In addition, the inclusion of normal colon epithelial cells provided preliminary insight into differential cellular responses to the investigated compounds and highlighted potential differences in selectivity profiles between Cucurbitacin B and Meleagrin. These findings may contribute to future translational investigations aimed at evaluating the therapeutic applicability and safety profiles of TYRO3-modulating natural compounds in CRC.

Nevertheless, several limitations of the present study should be acknowledged. Although the present study integrates complementary in vitro biological experiments with in silico computational analyses, all biological validation was performed using in vitro cell culture models, and no in vivo validation was included. Therefore, the translational relevance of these findings requires further confirmation in appropriate animal models and additional preclinical systems. Although modulation of TYRO3 expression was consistently observed, downstream signaling pathways implicated in TYRO3-mediated oncogenic activity, including PI3K/AKT, MAPK/ERK, and STAT-associated signaling, were not directly investigated. Future studies evaluating these pathways would provide important mechanistic insight into the relationship between TYRO3 modulation and the observed anticancer effects. Furthermore, TYRO3 protein expression was assessed exclusively by flow cytometry, which primarily reflects cell-surface-associated receptor levels rather than total cellular protein abundance. Complementary approaches such as Western blotting or immunofluorescence microscopy would provide additional information regarding total cellular TYRO3 expression, receptor localization, and subcellular distribution.

The computational analyses performed in the present study should also be interpreted within the context of their inherent limitations. Although molecular docking, molecular dynamics simulations, and MM-GBSA analyses collectively indicated stable and energetically favorable predicted interactions between the investigated compounds and TYRO3, these computational approaches are inherently predictive and cannot substitute for biophysical binding assays (e.g., surface plasmon resonance or isothermal titration calorimetry) or genetic/pharmacological target-validation studies. Therefore, direct target engagement remains to be experimentally confirmed. In addition, the pharmacokinetic and drug-likeness properties reported in this study were derived exclusively from the SwissADME computational platform, and no in vitro permeability (e.g., Caco-2 or PAMPA) or metabolic stability (e.g., liver microsomal) assays were performed. Consequently, the SwissADME-based drug-likeness assessment should be regarded as a theoretical, hypothesis-generating estimate rather than an experimentally validated pharmacokinetic profile.

Although the observed modulation of TYRO3 expression, together with the molecular docking and molecular dynamics findings, suggests a potential association between TYRO3 and the observed cellular responses, these data do not establish a direct causal relationship. Future studies employing TYRO3-specific knockdown, overexpression, or pharmacological inhibition approaches will be required to determine the relative contribution of TYRO3-dependent and TYRO3-independent mechanisms underlying the observed cellular responses.

Overall, the present study demonstrates that modulation of TYRO3 expression occurs concurrently with multifaceted anticancer effects induced by Cucurbitacin B and Meleagrin in CRC cells. Collectively, these findings further support the therapeutic relevance of TYRO3 as a molecular target warranting further mechanistic investigation in CRC and provide a rationale for further investigation of these compounds in preclinical CRC models.

## 4. Materials and Methods

### 4.1. Reagents and Antibodies

Cucurbitacin B (≥98% purity; Cat. No: 14820) and Meleagrin (Cat. No: 17850) were obtained from Cayman Chemical (Ann Arbor, MI, USA). Stock solutions were prepared in dimethyl sulfoxide (DMSO) (Sigma-Aldrich, St. Louis, MO, USA, Cat. No: 41640) and diluted to the desired concentrations in culture medium immediately prior to use.

Cell culture reagents included Dulbecco’s Modified Eagle Medium (DMEM, high glucose with L-glutamine and sodium pyruvate, Cat. No: DMEM-HPA) and phosphate-buffered saline (PBS, without Ca^2+^ and Mg^2+^; Cat. No: PBS-1A), both purchased from Capricorn Scientific (Ebsdorfergrund, Germany), and Eagle’s Minimum Essential Medium (EMEM; Cat. No: 30-2003) from the American Type Culture Collection (ATCC, Manassas, VA, USA). Fetal bovine serum (FBS) was obtained from PAN-Biotech (Aidenbach, Germany), and penicillin–streptomycin solution (Cat. No: PS-B) from Capricorn Scientific.

Human CRC cell lines HCT-116 (ATCC^®^ CCL-247™) and HT-29 (ATCC^®^ HTB-38™), as well as the normal human colon epithelial cell line CCD 841 CoN (ATCC^®^ CRL-1790™), were purchased from ATCC.

For gene expression analyses, total RNA was isolated using the PureLink™ RNA Mini Kit (Invitrogen, Thermo Fisher Scientific, Waltham, MA, USA, Cat. No: 12183020). cDNA synthesis was performed using the QuantiNova Reverse Transcription Kit (Qiagen, Hilden, Germany, Cat. No: 205411). RT-qPCR was carried out using TaqMan Gene Expression Assays for TYRO3 and *ACTB* together with TaqMan Gene Expression Master Mix (Applied Biosystems, Foster City, CA, USA).

TYRO3 protein expression was assessed using a mouse monoclonal anti-TYRO3 antibody (clone A-7; Cat. No: sc-166359; Santa Cruz Biotechnology, Dallas, TX, USA), followed by a fluorescein isothiocyanate (FITC)-conjugated secondary antibody (m-IgGκ BP-FITC; Cat. No: sc-516140; Santa Cruz Biotechnology).

Apoptosis was evaluated using an Annexin V-FITC/PI Apoptosis Detection Kit (Cat. No: E-CK-A211) and a Caspase-3/7 Activity Assay Kit (Cat. No: E-CK-A383) (Elabscience, Houston, TX, USA). Mitochondrial membrane potential was determined using the JC-1 assay kit (MitoPT™ JC-1 Assay Kit; ImmunoChemistry Technologies, Bloomington, MN, USA).

All other chemicals and reagents were of analytical grade and obtained from standard commercial suppliers.

### 4.2. Cell Culture

Human CRC cell lines HCT-116 and HT-29, as well as the normal human colon epithelial cell line CCD 841 CoN, were used in this study. HCT-116 and HT-29 cells were cultured in Dulbecco’s Modified Eagle Medium (DMEM) supplemented with 10% fetal bovine serum (FBS) and 1% penicillin–streptomycin, while CCD 841 CoN cells were maintained in Eagle’s Minimum Essential Medium (EMEM) supplemented with 10% FBS and 1% penicillin–streptomycin.

All cells were incubated at 37 °C in a humidified atmosphere containing 5% CO_2_. Cell morphology and growth were routinely monitored under an inverted microscope, and culture media were refreshed every 2–3 days.

Upon reaching approximately 80–90% confluency, cells were detached using trypsin–EDTA and subcultured into new culture flasks for subsequent experiments. Cells were used within limited passage numbers and were routinely monitored for potential contamination.

### 4.3. Cell Viability and Proliferation Analysis

#### 4.3.1. MTT Assay

Cell viability was evaluated using the MTT assay. HCT-116, HT-29, and CCD 841 CoN cells were seeded into 96-well plates at a density of 1 × 10^4^ cells per well in 200 µL of culture medium and incubated for 24 h to allow cell attachment.

Following incubation, cells were treated with various concentrations of Meleagrin and Cucurbitacin B and further incubated for 48 h. At the end of the treatment period, the culture medium was removed, and cells were washed with phosphate-buffered saline (PBS). Subsequently, 100 µL of fresh medium and 10 µL of MTT solution were added to each well, and the plates were incubated for 4 h at 37 °C.

After incubation, 100 µL of sodium dodecyl sulfate (SDS) solution was added to dissolve the formazan crystals, and the plates were incubated overnight. Absorbance was measured at 570 nm using a microplate reader (Biotek Instruments, Winooski, VT, USA).

Cell viability was calculated as a percentage relative to the untreated control group, which was considered 100%. All experiments were performed in triplicate and repeated at least three independent times. Experiments were performed under identical conditions across all groups.

Concentration–response curves were generated using GraphPad Prism (version 10.6.1 for macOS; GraphPad Software, San Diego, CA, USA), and IC_50_ values were calculated by nonlinear regression analysis using the log(inhibitor) versus normalized response (variable slope) model.

#### 4.3.2. Real-Time Cell Analysis (xCELLigence)

Cell proliferation was monitored in real time using the xCELLigence Real-Time Cell Analysis (RTCA) system (Acea Biosciences, San Diego, CA, USA), which monitors electrical impedance as an indicator of cell proliferation and viability.

For the analysis, 16-well E-plates were used. Initially, 100 µL of culture medium was added to each well, and background measurements were recorded. Subsequently, cells were seeded at a density of 1 × 10^4^ cells per well in 100 µL of medium. Plates were incubated at room temperature for 30 min to allow cell settling and then transferred to the RTCA device.

Cells were cultured under standard conditions (37 °C, 5% CO_2_), and impedance measurements were recorded every 15 min. After 24 h, when cells reached the logarithmic growth phase, the medium was replaced with fresh medium containing different concentrations of Meleagrin or Cucurbitacin B.

Cell index values were continuously monitored for up to 72 h. The data obtained were analyzed to evaluate the time- and dose-dependent effects of the compounds on cell proliferation.

### 4.4. Flow Cytometric Analysis of TYRO3 Expression

TYRO3 receptor protein expression in HCT-116 and HT-29 cells was analyzed by flow cytometry. Based on IC_50_ values obtained from proliferation assays, HCT-116 cells were treated with Meleagrin (5 and 10 µM) or Cucurbitacin B (5 and 15 µM), whereas HT-29 cells were treated with Meleagrin (3 and 5 µM) or Cucurbitacin B (10 and 20 µM). Concentrations were selected based on IC_50_ values and preliminary optimization studies designed to identify biologically active concentrations suitable for downstream mechanistic analyses.

Following treatment, cells were harvested, washed with phosphate-buffered saline (PBS), and resuspended at a density of approximately 5 × 10^5^ cells/mL. To minimize non-specific binding, cells were incubated in PBS containing 1% bovine serum albumin (BSA). An isotype-matched control antibody was used to evaluate non-specific background staining.

Cells were then incubated with a mouse monoclonal anti-TYRO3 primary antibody (clone A-7; Santa Cruz Biotechnology, Dallas, TX, USA) for 1 h at room temperature in the dark.

After washing with PBS, cells were incubated with a fluorescein isothiocyanate (FITC)-conjugated secondary antibody (diluted 1:1000 in PBS containing 1% BSA) for 15 min at room temperature in the dark. Following staining, cells were washed again and resuspended in PBS for analysis.

Flow cytometric acquisition was performed using a NovoCyte 2000R flow cytometer (ACEA Biosciences, San Diego, CA, USA). Cell populations were gated based on forward- and side-scatter (FSC/SSC) characteristics, and doublets were excluded prior to fluorescence analysis.

TYRO3 protein expression was quantified by comparing stained samples with the secondary-antibody-only control. Mean fluorescence intensity (MFI) values of gated singlet cell populations were calculated for quantitative assessment. Representative flow cytometric gating strategies are provided in Supplementary Figure S1.

### 4.5. RNA Isolation and RT-qPCR Analysis

Total RNA was isolated from HCT-116 and HT-29 cells following treatment with Meleagrin (3, 5, and 10 µM) or Cucurbitacin B (5 and 10 µM) for 24 h using the PureLink™ RNA Mini Kit (Invitrogen, Thermo Fisher Scientific, Waltham, MA, USA), according to the manufacturer’s instructions.

Briefly, cells were lysed in lysis buffer containing β-mercaptoethanol, and the lysates were homogenized. Ethanol was added to facilitate RNA binding to silica membrane columns. After sequential washing steps, total RNA was eluted in RNase-free water and stored at −80 °C until further use. RNA concentration and purity were determined spectrophotometrically, and RNA integrity was verified by agarose gel electrophoresis.

cDNA synthesis was performed using the QuantiNova Reverse Transcription Kit (Qiagen, Hilden, Germany) following the manufacturer’s protocol. Approximately 0.5–1 µg of total RNA was used for reverse transcription in a total reaction volume of 20 µL. The reaction was carried out at 42 °C for 60 min, followed by enzyme inactivation at 85 °C for 5 min.

To confirm successful cDNA synthesis, conventional PCR amplification of the housekeeping gene *ACTB* was performed using gene-specific primers, and PCR products were analyzed by agarose gel electrophoresis.

RT-qPCR was performed using TaqMan Gene Expression Assays (Applied Biosystems, Foster City, CA, USA) for TYRO3 and *ACTB*. Reactions were prepared in a total volume of 20 µL containing cDNA, gene-specific probe assays, and TaqMan Gene Expression Master Mix (Applied Biosystems).

Amplification was carried out using a Bio-Rad CFX96 real-time PCR detection system (Bio-Rad Laboratories, Hercules, CA, USA) under the following conditions: an initial denaturation step at 94 °C for 5 min, followed by 45 cycles of 94 °C for 10 s, 60 °C for 30 s, and 74 °C for 20 s.

Relative gene expression levels were calculated using the 2^−ΔΔCt^ method. TYRO3 expression levels were normalized to the housekeeping gene *ACTB* and expressed relative to the untreated control group.

### 4.6. Apoptosis Assays

#### 4.6.1. Annexin V-FITC/PI Staining

Apoptotic cell death was evaluated using Annexin V-FITC/propidium iodide (PI) staining followed by flow cytometric analysis. HCT-116 and HT-29 cells were treated with Meleagrin (5 and 10 µM) or Cucurbitacin B (5 and 15 µM) for 24 h. Hydrogen peroxide (H_2_O_2_) was used as a positive control.

Following treatment, both adherent and floating cells were collected, washed with phosphate-buffered saline (PBS), and resuspended at a density of approximately 1–5 × 10^5^ cells per sample. To minimize mechanical damage, cells were handled gently, and trypsinization was performed carefully.

Cells were resuspended in 500 µL of 1× Annexin V binding buffer. Subsequently, 5 µL of Annexin V-FITC and 5 µL of propidium iodide (PI) were added to each sample. Samples were gently mixed and incubated for 15 min at room temperature in the dark.

After incubation, samples were immediately analyzed by flow cytometry. Annexin V-FITC fluorescence was detected in the FITC channel, while PI fluorescence was detected in the PerCP channel. Cell populations were classified as viable, early apoptotic, late apoptotic, or necrotic based on Annexin V and PI staining patterns.

The percentage of Annexin V-positive cells was calculated and compared between treatment and control groups.

#### 4.6.2. Caspase-3/7 Activity Assay

Caspase-3/7 activity was measured using a commercial Caspase-3/7 Activity Assay Kit (Elabscience, Houston, TX, USA; Cat. No: E-CK-A383) according to the manufacturer’s instructions. The assay is based on the cleavage of a specific chromogenic substrate (Ac-DEVD-pNA) by active caspase-3 and caspase-7, resulting in the release of p-nitroaniline (pNA), which can be quantified spectrophotometrically.

HCT-116 and HT-29 cells were treated with Meleagrin (5 and 10 µM) or Cucurbitacin B (5 and 15 µM) for 24 h. Following treatment, cells were collected and lysed using the lysis buffer provided in the kit to obtain protein extracts.

Equal amounts of cell lysates were incubated with the caspase substrate according to the manufacturer’s protocol. After incubation, the release of pNA was measured at 405 nm using a microplate reader.

Caspase-3/7 activity was calculated based on absorbance values and expressed relative to the untreated control group.

#### 4.6.3. Mitochondrial Membrane Potential (JC-1 Assay)

Mitochondrial membrane potential (ΔΨm) was assessed using a JC-1 assay kit (MitoPT™ JC-1 Assay Kit, ImmunoChemistry Technologies, Bloomington, MN, USA) according to the manufacturer’s instructions.

HCT-116 and HT-29 cells were seeded into 6-well plates at a density of 1 × 10^6^ cells/mL and incubated for 24 h. Cells were then treated with Meleagrin (5 and 10 µM) or Cucurbitacin B (5 and 15 µM) for 24 h.

Following treatment, cells were harvested by trypsinization, washed with phosphate-buffered saline (PBS), and centrifuged at 8000 rpm for 5 min. The cell pellet was resuspended in 1 mL of 1× JC-1 staining solution and incubated at 37 °C for 15 min in the dark.

After incubation, cells were washed twice with assay buffer and centrifuged at 8000 rpm for 5 min. The final cell pellet was resuspended in assay buffer, and fluorescence measurements were performed.

JC-1 aggregates (red fluorescence) and monomers (green fluorescence) were detected using appropriate excitation/emission settings. The ratio of red to green fluorescence intensity was calculated as an indicator of mitochondrial membrane potential.

### 4.7. Cell Migration Assay

Cell migration was evaluated using a wound healing (scratch) assay. HCT-116 and HT-29 cells were seeded into 24-well plates and allowed to grow to a confluent monolayer.

To synchronize the cells, the culture medium was replaced with serum-free medium, and cells were incubated for an additional 24 h. A linear scratch was created across the cell monolayer using a sterile 100 µL pipette tip. Detached cells were removed by washing the wells with phosphate-buffered saline (PBS).

Cells were then incubated with culture medium containing 1% fetal bovine serum (FBS) and treated with different concentrations of Meleagrin or Cucurbitacin B. For HCT-116 cells, Meleagrin (5 and 10 µM) and Cucurbitacin B (5 and 15 µM) were used, whereas for HT-29 cells, Meleagrin (3 and 5 µM) and Cucurbitacin B (10 and 20 µM) were applied.

Images of the wound area were captured at 0, 24, and 48 h using an inverted microscope (Olympus Corporation, Tokyo, Japan). Cell migration was quantified by measuring the wound area at each time point, and the percentage of remaining wound area was calculated relative to the initial wound area.

### 4.8. Statistical Analysis

All data are presented as mean ± standard deviation (SD). Statistical analyses were performed using GraphPad Prism software (GraphPad Software, San Diego, CA, USA).

Comparisons among groups were performed using one-way analysis of variance (ANOVA) followed by appropriate post hoc multiple comparison tests. A *p*-value < 0.05 was considered statistically significant.

Data are presented as mean ± SD. Statistical significance was evaluated relative to untreated control cells and defined as * *p* < 0.05, ** *p* < 0.01, and *** *p* < 0.001.

### 4.9. In Silico Molecular Docking and ADME Analysis

An initial virtual screening workflow using iGEMDOCK was performed to identify candidate TYRO3-modulating compounds from the investigated natural compound library. Based on docking performance and experimental accessibility, Cucurbitacin B and Meleagrin were selected for subsequent docking analyses and biological experiments. The crystallographic structure of the TYRO3 kinase domain (PDB ID: 3QUP) was retrieved from the Protein Data Bank and used for refined molecular docking analyses.

Protein preparation was performed using UCSF Chimera (version 1.17.3). The co-crystallized ligand and crystallographic water molecules were removed, hydrogen atoms were added, and partial charges were assigned prior to docking calculations. Ligand structures were obtained from the PubChem database and geometrically optimized before analysis.

Molecular docking simulations were conducted using AutoDock Vina (version 1.1.2) interfaced with UCSF Chimera (version 1.17.3) via the ViewDock module. The binding site was defined based on the position of the co-crystallized inhibitor within the catalytic pocket. Docking poses were evaluated based on predicted binding affinity and protein–ligand interaction profiles. Interaction analyses and visualization were performed using BIOVIA Discovery Studio Visualizer.

To validate the docking protocol, the co-crystallized inhibitor was re-docked into the TYRO3 kinase domain, and the root-mean-square deviation (RMSD) between the docked and crystallographic conformations was calculated using DockRMSD. RMSD values ≤ 2.0 Å were considered indicative of successful reproduction of the native binding mode.

Pharmacokinetic and drug-likeness properties of the investigated compounds were evaluated using the SwissADME web tool. Gastrointestinal absorption, Lipinski compliance, P-glycoprotein substrate prediction, and medicinal chemistry filters were analyzed using the SMILES representations of the compounds. Detailed docking parameters, ADME analyses, and additional molecular dynamics data are provided in Supplementary File S2. The molecular dynamics simulations were performed using the computational resources provided by the TÜBİTAK ULAKBİM High Performance and Grid Computing Center (TRUBA).

## 5. Conclusions

In conclusion, the present study demonstrates that the natural compounds Cucurbitacin B and Meleagrin exert promising anticancer effects in CRC cells while also modulating TYRO3 expression. Both compounds inhibited cell proliferation and migration and promoted apoptotic cell death, whereas Cucurbitacin B produced more pronounced antiproliferative and pro-apoptotic effects. In contrast, Meleagrin displayed comparatively greater selectivity toward CRC cells than normal colon epithelial cells. In the absence of TYRO3-specific gain- or loss-of-function experiments, the present findings should be interpreted as evidence of an association between TYRO3 modulation and the observed biological responses, rather than as definitive proof of a direct causal or mechanistic role for TYRO3. The observed modulation of TYRO3 at both mRNA and protein levels further supports the rationale for investigating TYRO3 as a potential therapeutic target in CRC. Further mechanistic studies are required to determine the causal contribution of TYRO3 modulation to the observed anticancer effects. Collectively, our complementary in vitro and in silico findings provide a rationale for further mechanistic studies and preclinical evaluation of TYRO3-modulating natural compounds in colorectal cancer, including validation in appropriate in vivo models to establish their therapeutic potential.

## Data Availability

The datasets generated and/or analyzed during the current study are available from the corresponding author upon reasonable request.

## Supplementary Materials

The following supporting information can be downloaded at: https://www.mdpi.com/article/10.3390/ijms27156759/s1. References [45,52,53] are cited in the Supplementary Materials.

## Abbreviations

The following abbreviations are used in this manuscript:

*ACTB*	Beta-actin
ADME	Absorption, distribution, metabolism, and excretion
AKT	Protein kinase B
AXL	AXL receptor tyrosine kinase
BSA	Bovine serum albumin
CRC	Colorectal cancer
CuB	Cucurbitacin B
DMEM	Dulbecco’s Modified Eagle Medium
DMSO	Dimethyl sulfoxide
EGFR	Epidermal growth factor receptor
EMEM	Eagle’s Minimum Essential Medium
EMT	Epithelial–mesenchymal transition
ERK	Extracellular signal-regulated kinase
FBS	Fetal bovine serum
FITC	Fluorescein isothiocyanate
FSC/SSC	Forward scatter/side scatter
MAPK	Mitogen-activated protein kinase
MERTK	MER proto-oncogene tyrosine kinase
MFI	Mean fluorescence intensity
MMP	Mitochondrial membrane potential
MTT	3-(4,5-dimethylthiazol-2-yl)-2,5-diphenyltetrazolium bromide
PBS	Phosphate-buffered saline
PDB	Protein Data Bank
PI	Propidium iodide
PI3K	Phosphoinositide 3-kinase
RMSD	Root-mean-square deviation
ROS	Reactive oxygen species
RTCA	Real-time cell analysis
RT-qPCR	Real-time quantitative polymerase chain reaction
SD	Standard deviation
STAT3	Signal transducer and activator of transcription 3
TAM	TYRO3, AXL, and MERTK receptor family
TYRO3	TYRO3 protein tyrosine kinase receptor
