# Supplementary material for: Anticancer Effects of Cucurbitacin B and Meleagrin Associated with TYRO3 Downregulation in Colorectal Cancer Cells
Source: Int J Mol Sci. 2026 Jul 28;27(15):6759. doi: 10.3390/ijms27156759 (PMC13467144; doi:10.3390/ijms27156759)
Supplement: Supplementary file 1 [file ijms-27-06759-s001.zip › File S1.pdf]

Supplementary File S1. Supplementary Flow Cytometric Analysis of TYRO3 Expression

The following supplementary figures provide representative flow cytometric gating strategies used for the analysis of TYRO3 expression in colorectal cancer cell lines.

Figure S1

A. FSC/SSC gating

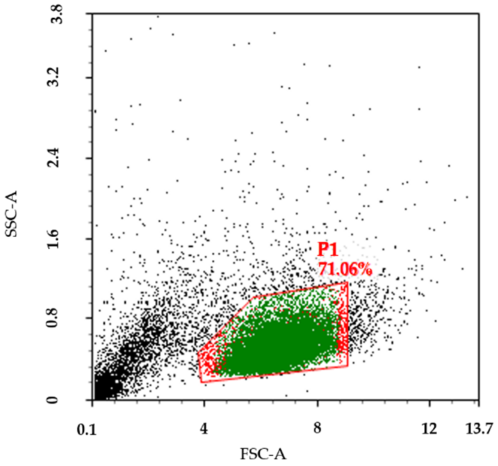

B. FSC-A/FSC-H singlet gating

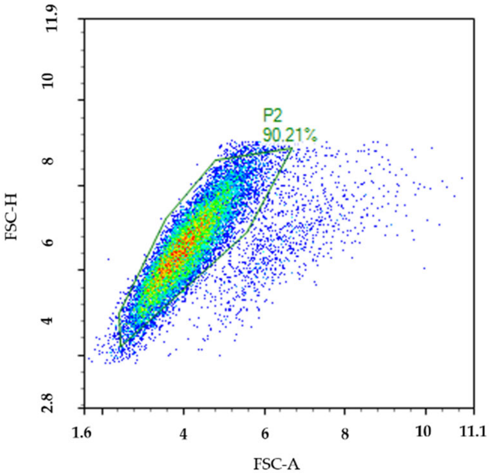

C. Isotype vs TYRO3 histogram

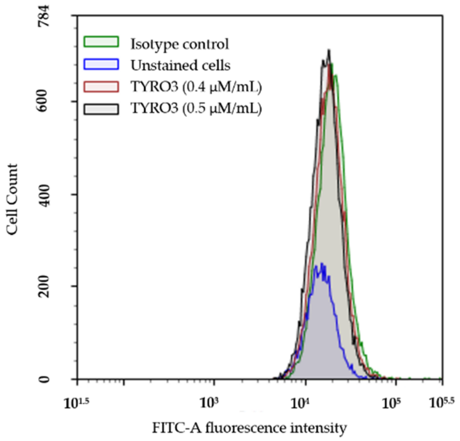

(A) HT-29. Representative flow cytometric gating strategy employed for the analysis of TYRO3 expression in HT-29 colorectal cancer cells.

A. FSC/SSC gating

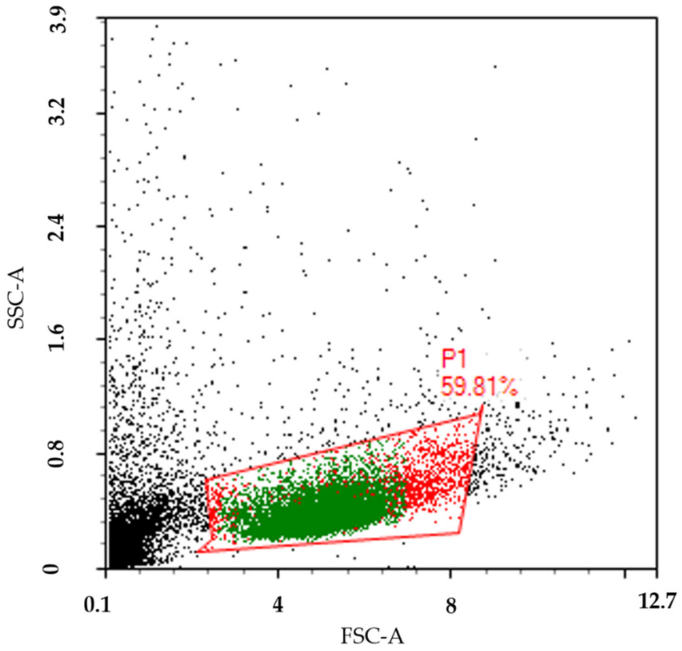

B. Singlet gating

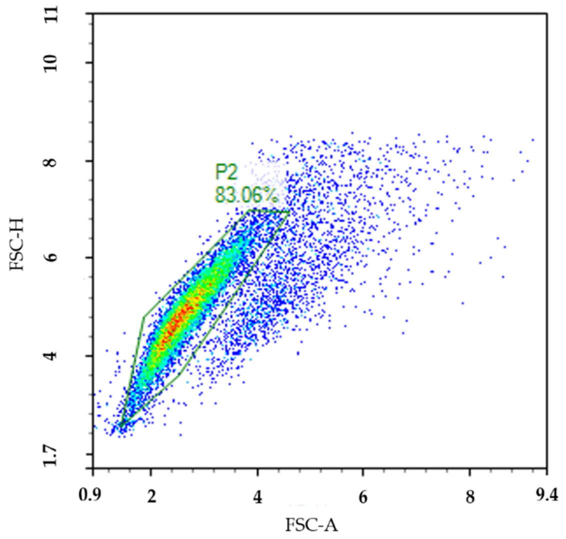

C. Isotype vs TYRO3 histogram

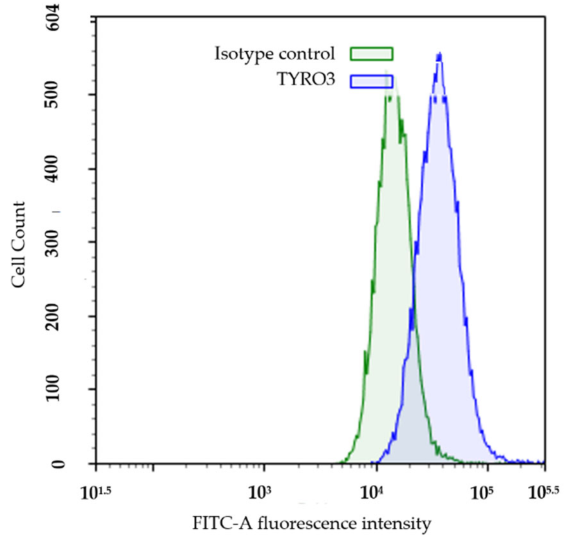

(B) HCT-116. Representative flow cytometric gating strategy employed for the analysis of TYRO3 expression in HCT-116 colorectal cancer cells.

Table S1. IC_{50} values of Meleagrin and Cucurbitacin B in HCT-116, HT-29 and CCD 841 CoN cells.

Compound	HCT-116 (μM)	HT-29 (μM)	CCD 841 CoN (μM)
Cucurbitacin B	5.83	16.79	49.42
Meleagrin	6.96	3.54	49.73

IC_{50} values were calculated by nonlinear regression analysis using the log(inhibitor) versus normalized response (variable slope) model in GraphPad Prism software.
