# Supplementary material for: Anticancer Effects of Cucurbitacin B and Meleagrin Associated with TYRO3 Downregulation in Colorectal Cancer Cells
Source: Int J Mol Sci. 2026 Jul 28;27(15):6759. doi: 10.3390/ijms27156759 (PMC13467144; doi:10.3390/ijms27156759)
Supplement: Supplementary file 1 [file ijms-27-06759-s001.zip › File S2.pdf]

Supplementary File S2. Computational Analyses

S2.1. Molecular Docking and Validation

Molecular docking simulations were conducted using AutoDock Vina, interfaced with UCSF Chimera (version 1.17.3) via the ViewDock module. The exhaustiveness parameter was set to 10 to ensure thorough conformational sampling of the binding site. The binding site was defined based on the position of the co-crystallized inhibitor within the catalytic pocket. The grid box was centered at x = 10.65, y = −9.85, and z = 13.47 Å, with dimensions of 16 × 16 × 16 Å, ensuring full coverage of the active site region. Docking poses were evaluated based on predicted binding affinity and protein–ligand interaction profiles. Protein–ligand interaction analysis and visualization of the resulting binding poses were performed using BIOVIA Discovery Studio (v24.1.0.23298, Dassault Systèmes, 2023) [1].

To validate the docking protocol, a redocking procedure was performed using the co-crystallized inhibitor. The root-mean-square deviation (RMSD) between the docked pose and the crystallographic conformation was calculated using DockRMSD [2], which performs atom mapping through a graph isomorphism approach, particularly suitable for symmetric molecules. An RMSD value ≤ 2.0 Å is generally accepted as indicative of successful reproduction of the crystallographic binding mode in docking validation studies [3].

Redocking of the co-crystallized inhibitor reproduced the experimental binding mode with an RMSD value of 1.240 Å, confirming the reliability of the docking protocol for subsequent analyses.

Table S2. Molecular Docking Parameters

**Parameter**	**Value**
**Docking software**	AutoDock Vina
**Interface**	UCSF Chimera 1.17.3
**Visualization software**	BIOVIA Discovery Studio
**RMSD software**	DockRMSD
**Grid center x**	10.65
**Grid center y**	−9.85
**Grid center z**	13.47
**Grid dimensions**	16 × 16 × 16 Å
**Exhaustiveness**	10
**RMSD value**	1.240 Å
**Acceptance criterion**	RMSD ≤ 2.0 Å

S2.2. ADME and Drug-Likeness Prediction

The pharmacokinetic properties and drug-likeness characteristics of the selected compounds were predicted using the SwissADME web server. Parameters including molecular weight, lipophilicity (LogP), topological polar surface area (TPSA), gastrointestinal absorption, blood–brain barrier permeability, water solubility, and compliance with Lipinski's rule of five were evaluated. The generated predictions were used to estimate the oral drug-likeness and pharmacokinetic suitability of the identified TYRO3-modulating compounds.

Table S3. Predicted ADME and drug-likeness properties of the selected compounds

Parameter	Meleagrin	Cucurbitacin B
Physicochemical Properties		
Molecular formula	$C_{23}H_{23}N_5O_4$	$C_{32}H_{46}O_8$
Molecular weight (g/mol)	433.46	558.70
Consensus Log P	0.95	3.10
TPSA (Å^2)	110.79	138.20
#H-bond donors	3	3
#H-bond acceptors	5	8
#Rotatable bonds	4	6
Fraction Csp3	0.26	0.75
Molar refractivity	127.33	150.94
Solubility		
Water solubility (ESOL class)	Moderately soluble	Moderately soluble
ESOL Log S (log mol/L)	−4.43	−4.57
log Kp — skin permeability (cm/s)	−6.79	−7.83
Absorption and Distribution		
GI absorption	High	Low
BBB permeant	No	No
P-gp substrate	Yes	Yes
Drug-Likeness Filters		
Lipinski violations	0	1 (MW > 500)
Ghose violations	0	3
Veber violations	0	0
Egan violations	0	1
Muegge violations	0	0
Bioavailability Score	0.55	0.55
Medicinal Chemistry		
PAINS alerts	0	0
Brenk alerts	2	2
Leadlikeness violations	1	1
Synthetic accessibility score	5.39	6.79

BBB, blood–brain barrier; GI, gastrointestinal; TPSA, topological polar surface area.

SwissADME was used to predict physicochemical characteristics, absorption-related parameters, drug-likeness properties, and medicinal chemistry descriptors of Meleagrin and Cucurbitacin B. Predictions were generated using the canonical SMILES representations of each compound. Abbreviations and parameter definitions are provided below.

Abbreviations: BBB = blood–brain barrier; ESOL = estimated solubility; GI = gastrointestinal; log Kp = skin permeability coefficient; Log P = octanol–water partition coefficient; Log S = aqueous solubility; MR = molar refractivity; PAINS = pan-assay interference compounds; P-gp = P-glycoprotein; TPSA = topological polar surface area.

Brenk alerts indicate the presence of structural features that may require consideration during future medicinal chemistry optimization.

Meleagrin complied with all evaluated drug-likeness filters, whereas Cucurbitacin B exhibited several violations, primarily associated with its higher molecular weight and physicochemical complexity.

S2.3. Molecular Dynamics Simulations and MM-GBSA Analyses

Additional molecular dynamics analyses were performed to further evaluate the stability of TYRO3–ligand complexes and to complement the primary RMSD and MM-GBSA results presented in the main manuscript. These analyses included RMSF, radius of gyration (Rg), solvent-accessible surface area (SASA), protein–ligand contact analyses, center-of-mass distance measurements, hydrogen-bond occupancy analyses, and residue-wise MM-GBSA free energy decomposition.

Additional molecular dynamics analyses of TYRO3 complexes with Cucurbitacin B and Meleagrin.

This supplementary figure provides additional molecular dynamics analyses supporting the stability of TYRO3–ligand complexes, including protein stability metrics, ligand stability analyses, residue-wise MM-GBSA decomposition profiles, and a summary of key simulation parameters.

A. Per-residue RMSF: Apo vs Holo Tyro3 (CuB)

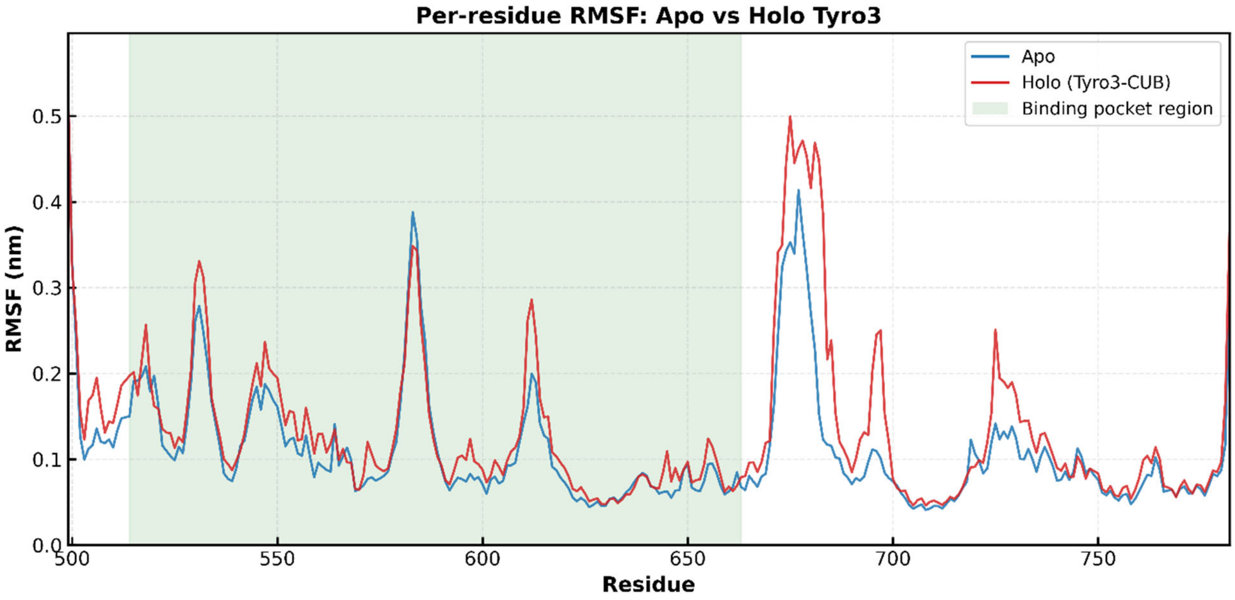

B. Per-residue RMSF: Apo vs Holo Tyro3 (Mel)

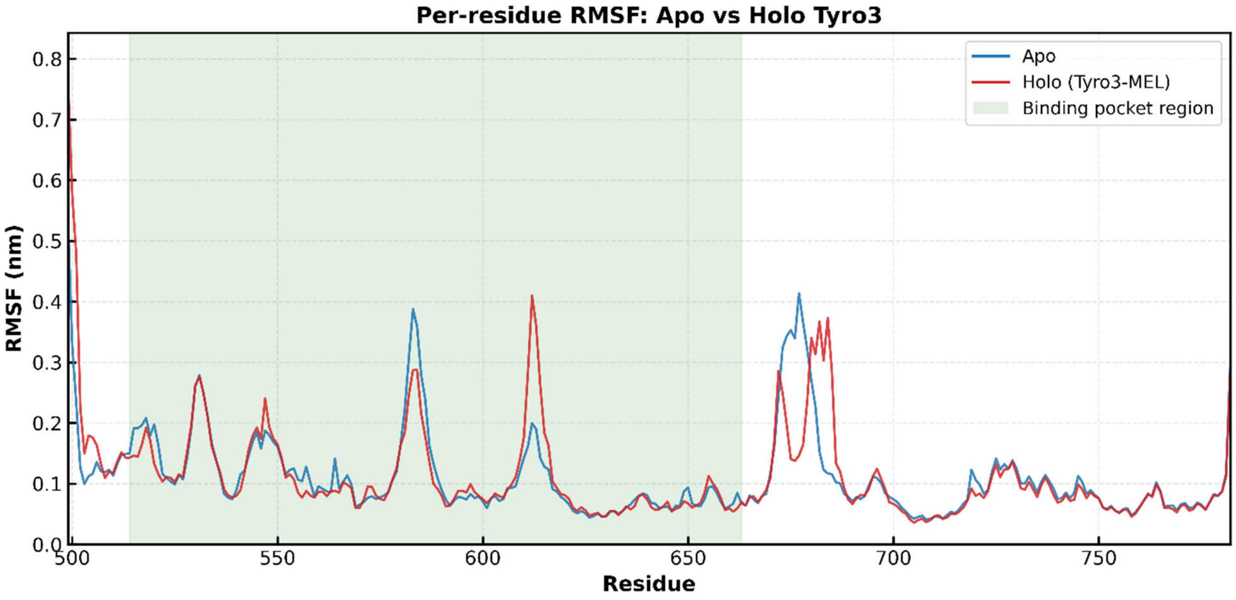

C. Radius of Gyration: Apo vs Holo Tyro3 (CuB)

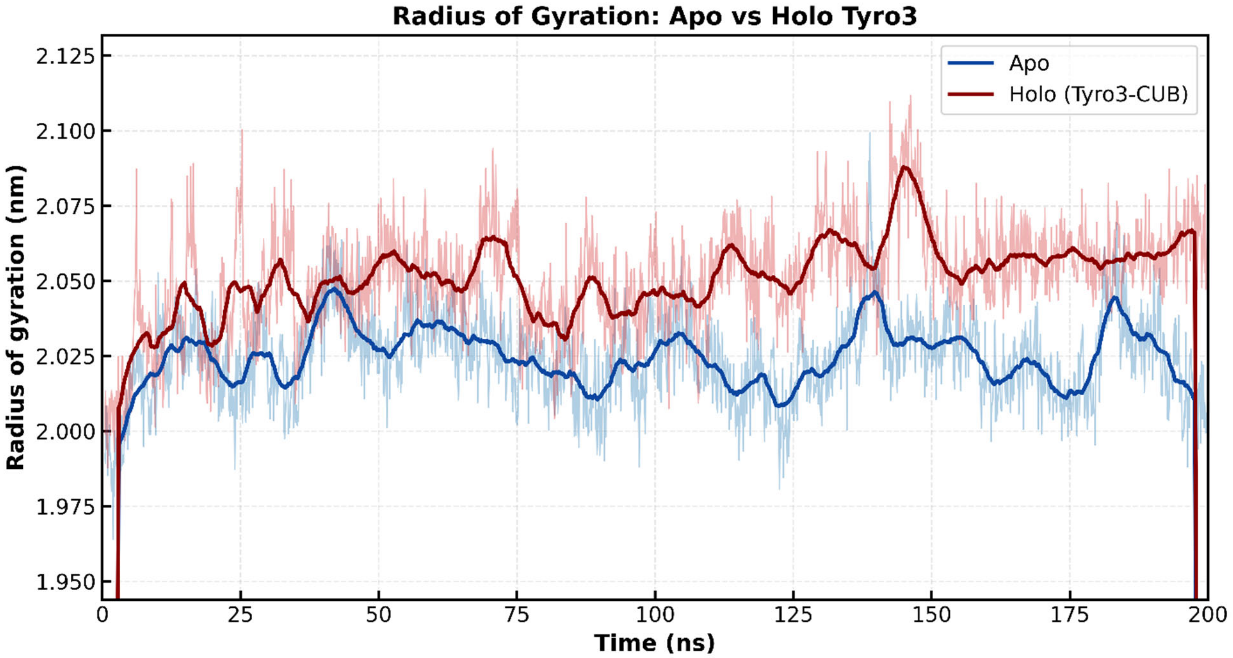

D. Radius of Gyration: Apo vs Holo Tyro3 (Mel)

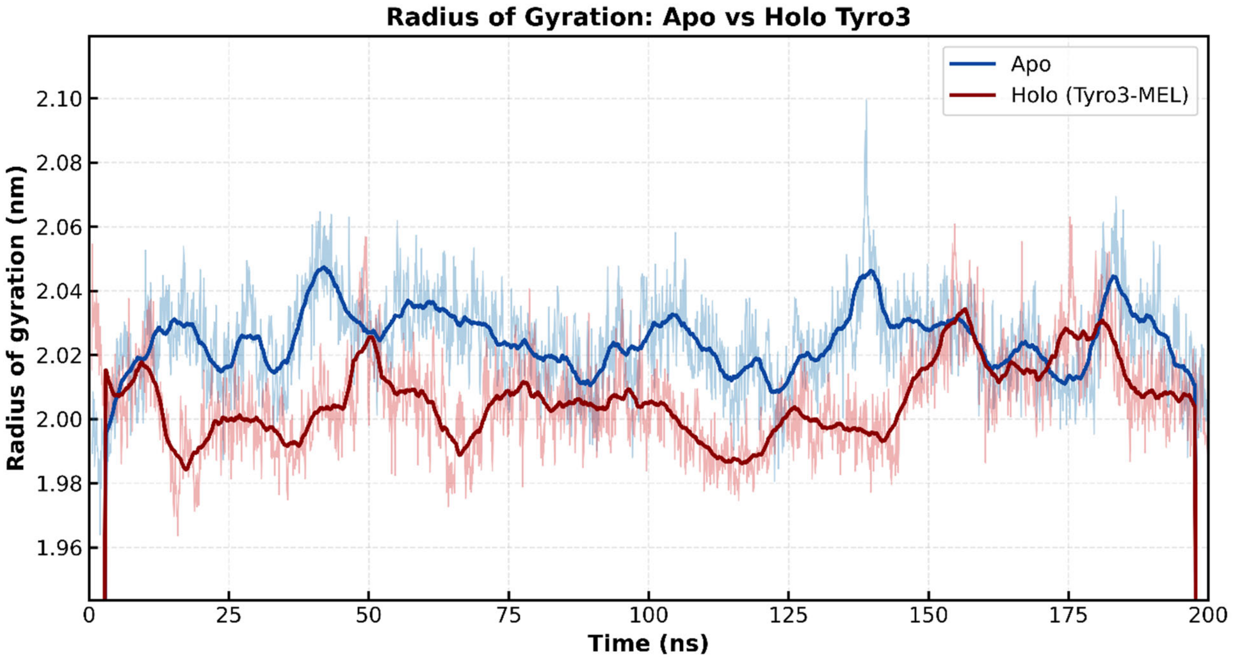

E. Solvent Accessible Surface Area: Apo vs Holo Tyro3 (CuB)

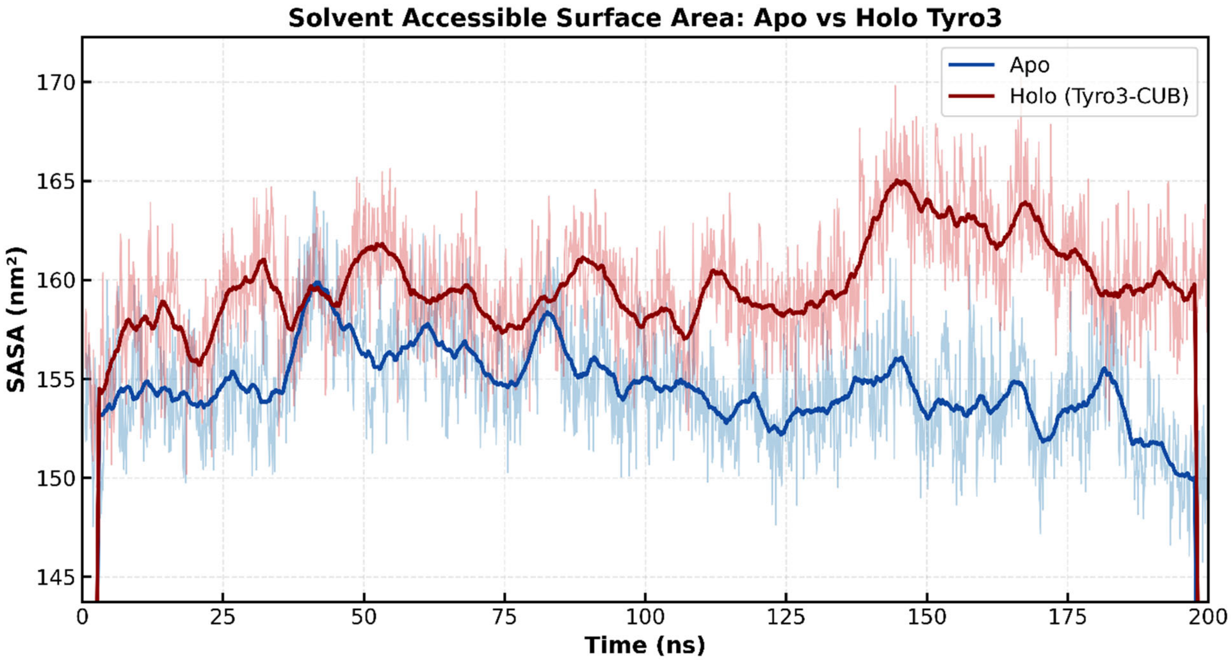

F. Solvent Accessible Surface Area: Apo vs Holo Tyro3 (Mel)

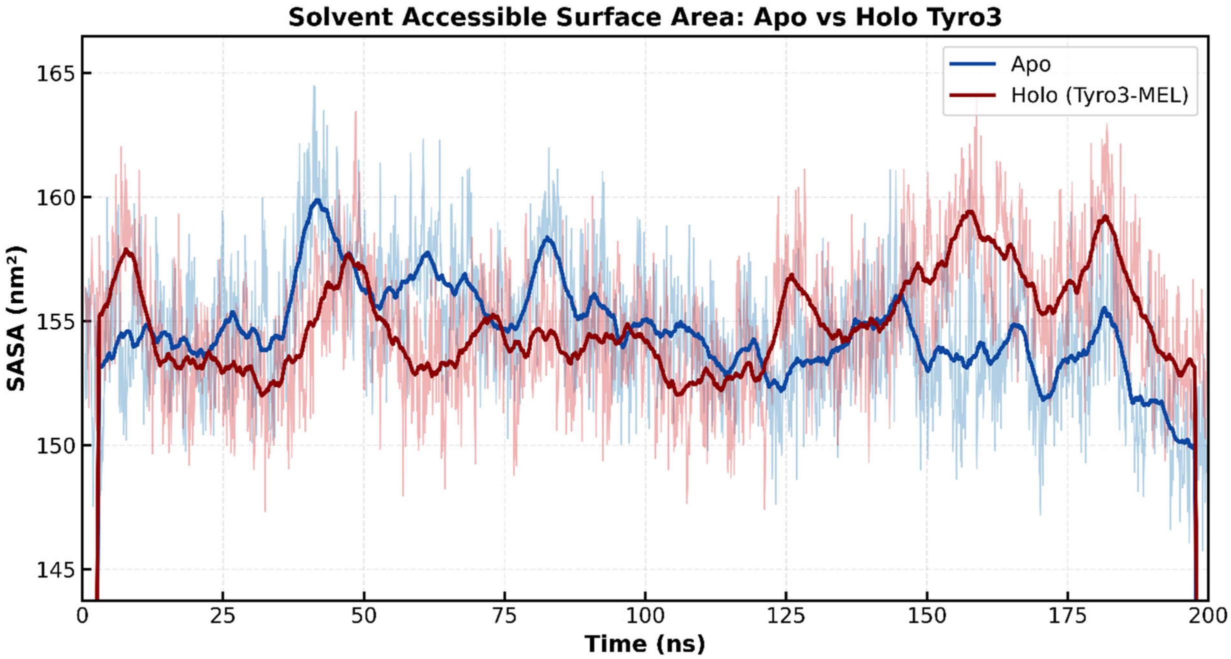

Figure S2A. Additional molecular dynamics analyses of TYRO3 complexes with Cucurbitacin B and Meleagrin.

(A) Per-residue root-mean-square fluctuation (RMSF) profiles of apo TYRO3 and the TYRO3–Cucurbitacin B complex. (B) RMSF profiles of apo TYRO3 and the TYRO3–Meleagrin complex. (C) Radius of gyration

(Rg) analysis of apo TYRO3 and the TYRO3–Cucurbitacin B complex. (D) Radius of gyration (Rg) analysis of apo TYRO3 and the TYRO3–Meleagrin complex. (E) Solvent-accessible surface area (SASA) profiles of apo TYRO3 and the TYRO3–Cucurbitacin B complex. (F) SASA profiles of apo TYRO3 and the TYRO3–Meleagrin complex during 200 ns molecular dynamics simulations.

Additional molecular dynamics analyses demonstrated that both TYRO3–ligand complexes maintained overall structural stability throughout the simulation period. RMSF, Rg, and SASA analyses revealed only modest differences between apo and holo systems, suggesting that ligand binding did not induce major structural perturbations in TYRO3. Rg and SASA profiles remained relatively stable over time, suggesting that ligand binding did not substantially alter the global compactness or solvent accessibility of TYRO3. These findings further support the structural stability of both TYRO3–ligand complexes observed in the primary molecular dynamics analyses.

G. Protein-CuB Contacts (TYRO3)

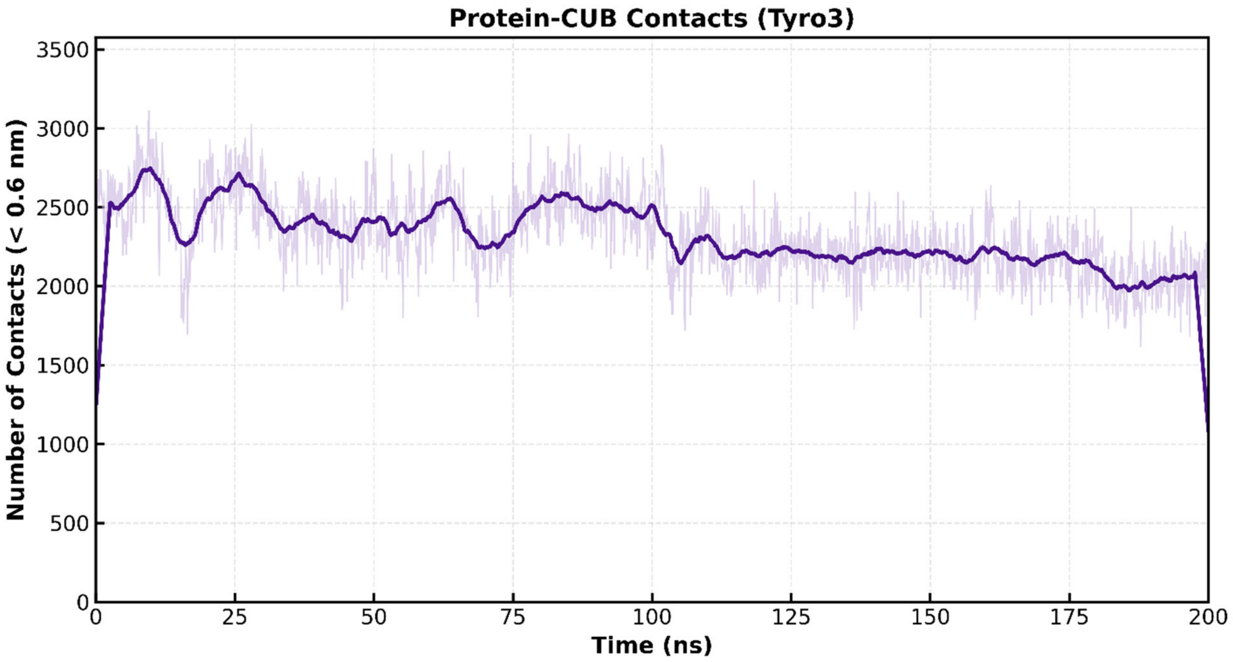

H. Protein-Mel Contacts (TYRO3)

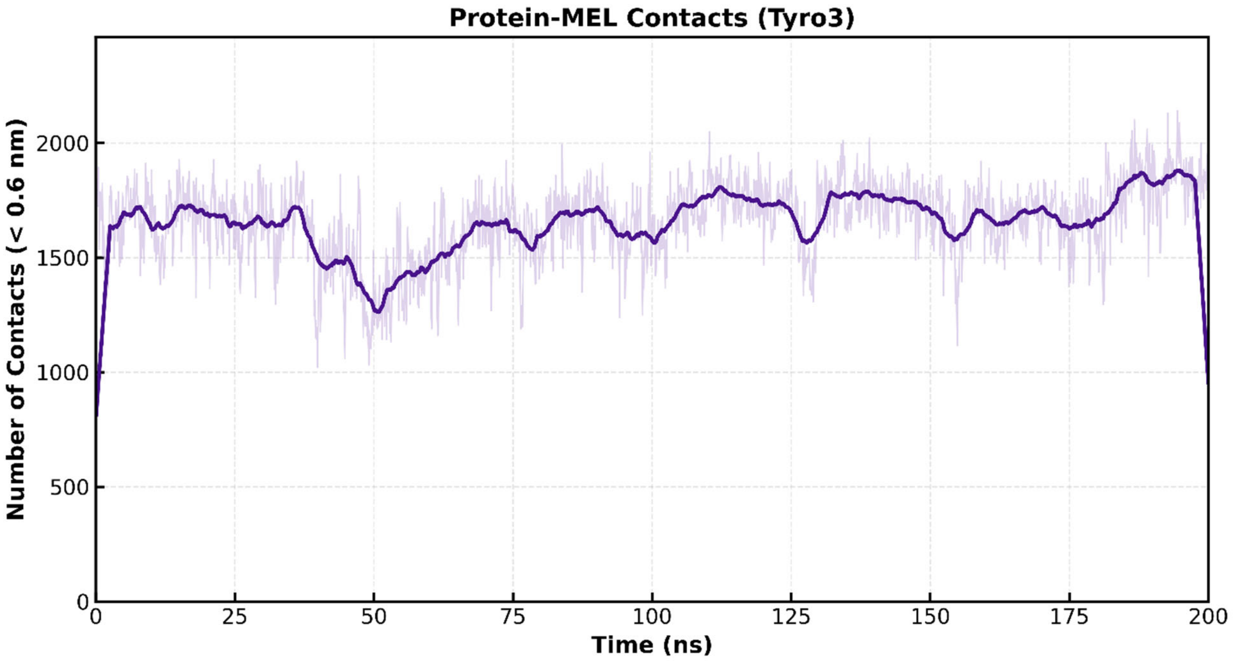

I. Center of Mass Distance: Protein-CuB (TYRO3)

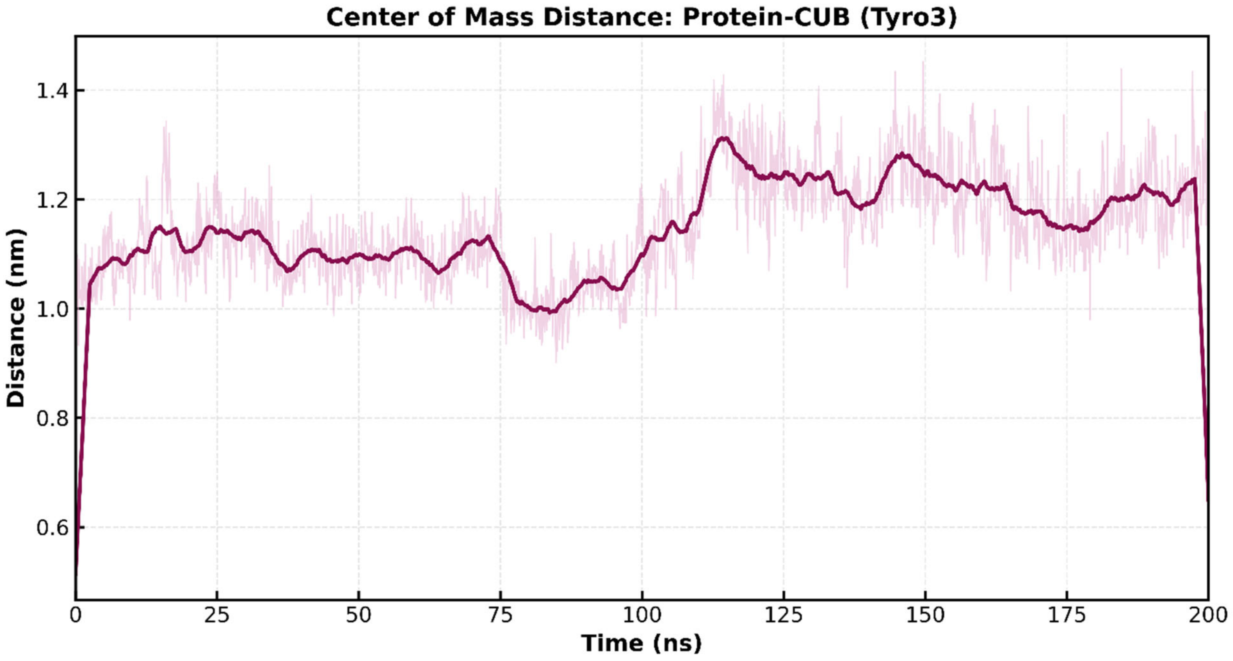

J. Center of Mass Distance: Protein-Mel (TYRO3)

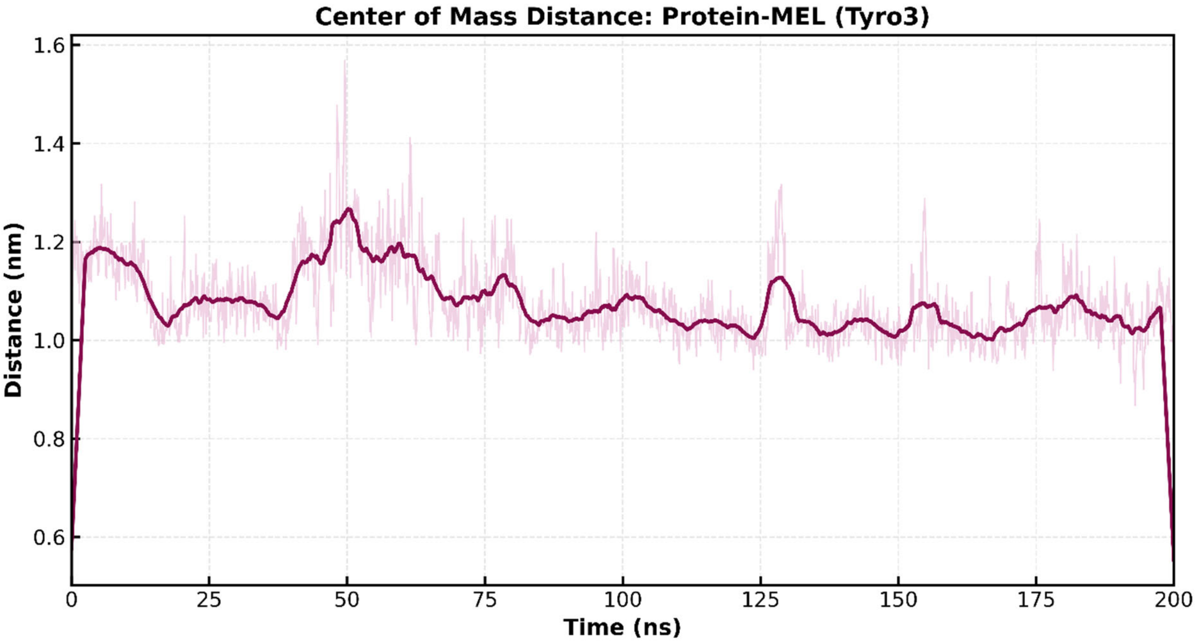

K. Hydrogen-bond occupancy analyses of TYRO3–CuB complexes

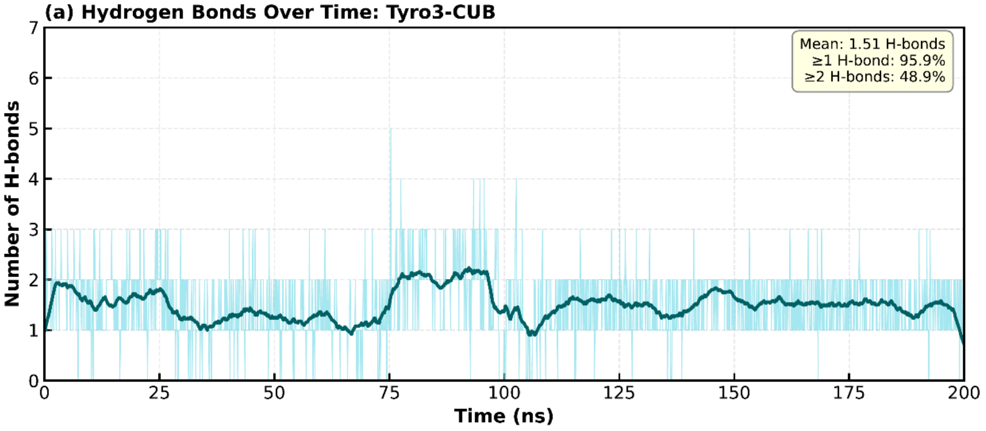

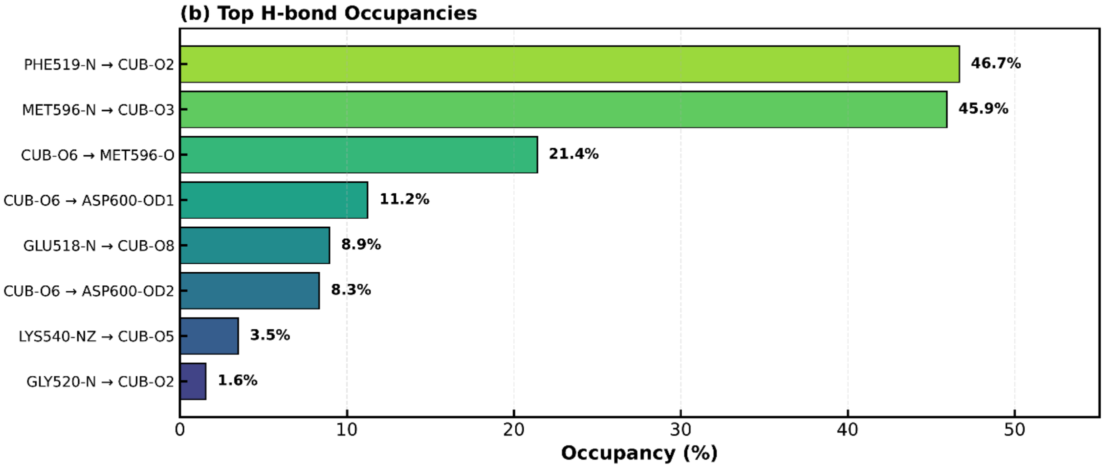

L. Hydrogen-bond occupancy analyses of TYRO3–Meleagrin complexes

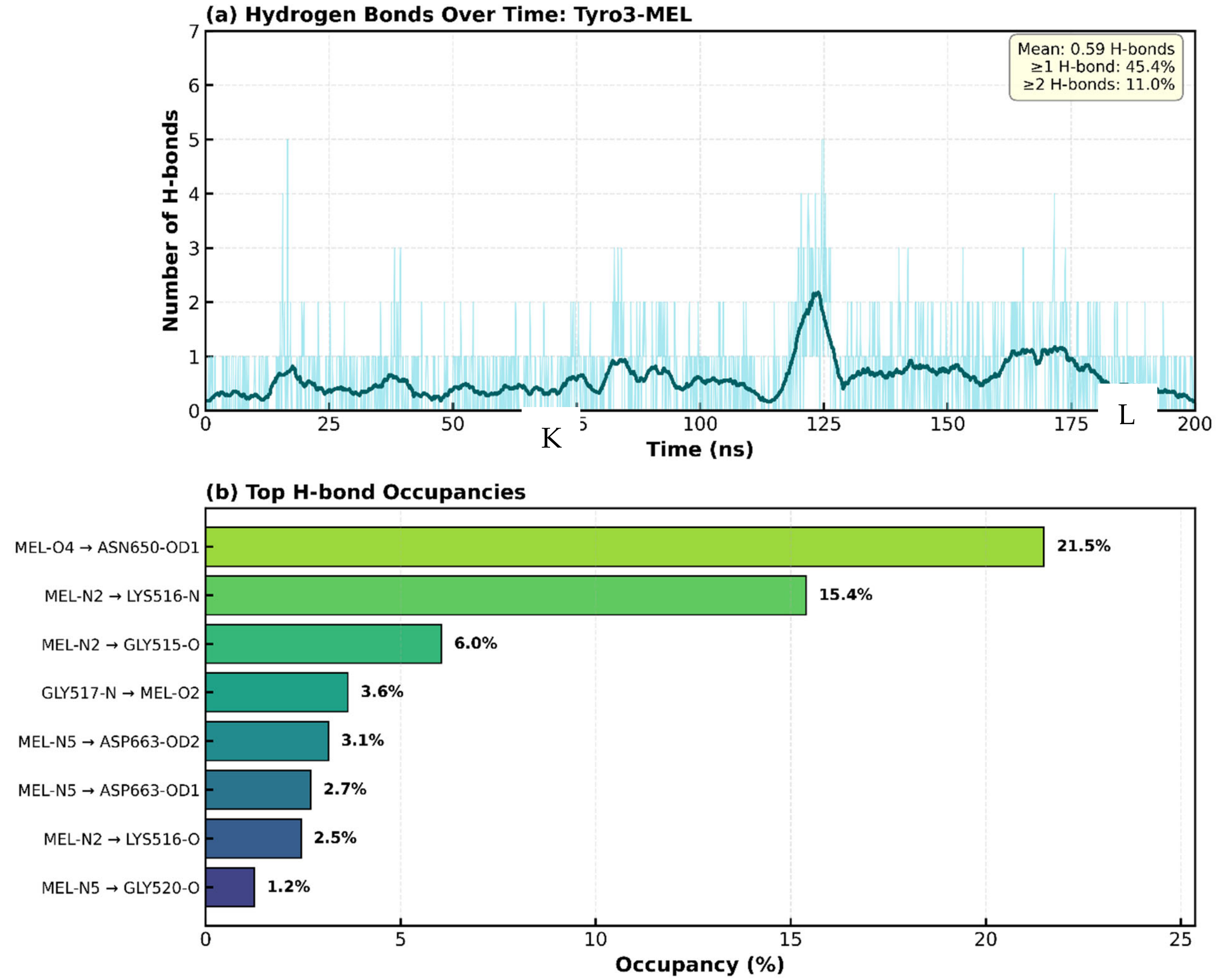

Figure S2B. Additional ligand stability analyses of TYRO3 complexes with Cucurbitacin B and Meleagrin.

(G–H) Protein–ligand contact number analyses of the TYRO3–Cucurbitacin B and TYRO3–Meleagrin complexes, respectively. (I–J) Center-of-mass (COM) distance profiles between TYRO3 and the bound ligand throughout the 200 ns simulation period. (K–L) Hydrogen-bond occupancy analyses of TYRO3–Cucurbitacin B and TYRO3–Meleagrin complexes. These analyses provide additional information regarding ligand retention, binding stability, and intermolecular interactions during molecular dynamics simulations.

Additional ligand-focused analyses further supported stable binding of both compounds within the TYRO3 binding pocket. Protein–ligand contact analyses demonstrated persistent interactions throughout the simulations, while COM distance profiles remained relatively stable over time, indicating sustained ligand retention within the binding site. Hydrogen-bond analyses revealed continuous intermolecular interactions, with Cucurbitacin B exhibiting a greater average number

of hydrogen bonds and higher hydrogen-bond occupancy than Meleagrin. Collectively, these findings further support the stable association of both compounds with TYRO3 during the 200 ns simulation period.

M. Per-residue Energy Decomposition (MM-GBSA) – TYRO3-CuB

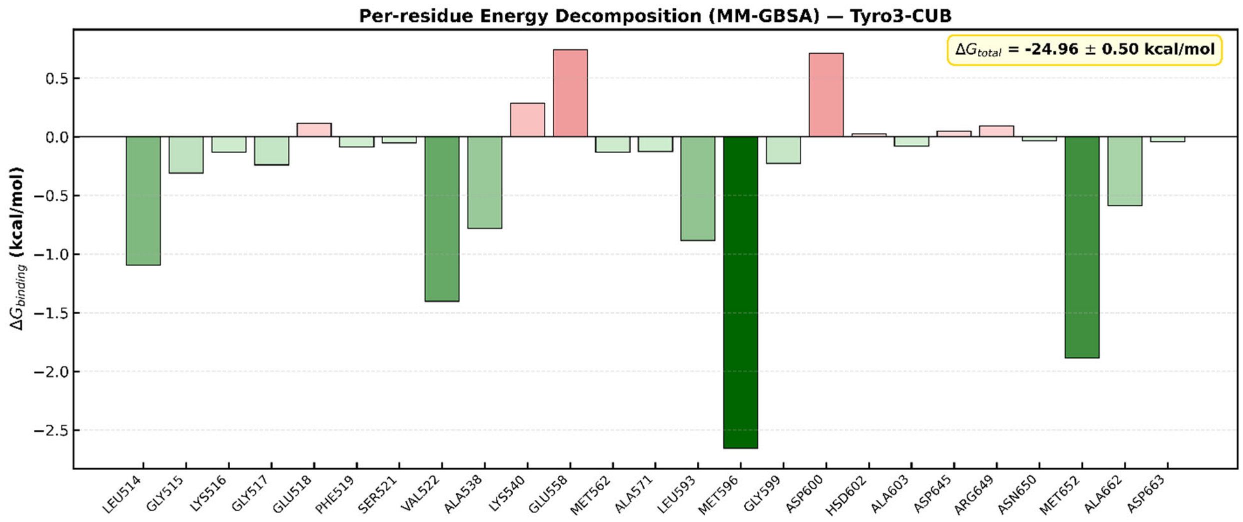

N. Per-residue Energy Decomposition (MM-GBSA) – TYRO3-Mel

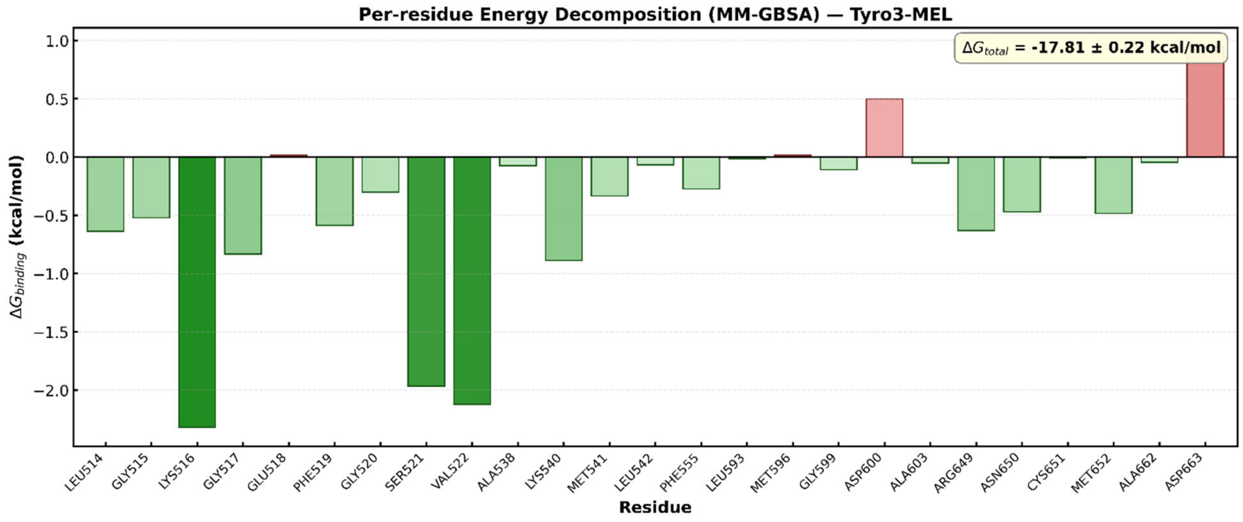

Figure S2C. Residue-wise MM-GBSA energy decomposition analyses of TYRO3 complexes with Cucurbitacin B and Meleagrin.

(M) Per-residue MM-GBSA free energy decomposition profile of the TYRO3–Cucurbitacin B complex calculated from the final 100 ns of the molecular dynamics trajectory. (N) Per-residue MM-GBSA free energy decomposition profile of the TYRO3–Meleagrin complex. Negative values indicate favorable

energetic contributions to ligand binding, whereas positive values indicate unfavorable contributions. The analyses identify key amino acid residues contributing to stabilization of the ligand–TYRO3 complexes.

Residue-wise MM-GBSA decomposition analyses identified several amino acid residues that contributed substantially to ligand stabilization within the TYRO3 binding pocket. In the TYRO3–Cucurbitacin B complex, favorable energetic contributions were predominantly associated with residues surrounding the ATP-binding region, including MET596 and neighboring hydrophobic residues. In contrast, the TYRO3–Meleagrin complex displayed a distinct energetic contribution profile, with LYS516, SER521, and VAL522 among the major contributors to binding stability. These findings further support the notion that Cucurbitacin B and Meleagrin interact with TYRO3 through partially distinct molecular interaction patterns.

Table S4. Summary of molecular dynamics simulation parameters and binding stability metrics for TYRO3 complexes with Cucurbitacin B and Meleagrin.

Parameter	Tyro3-CUB	Tyro3-MEL
Backbone RMSD (nm)	0.236 ± 0.037	0.205 ± 0.032
Ligand RMSD (nm)	0.547 ± 0.101	0.276 ± 0.091
COM distance (nm)	1.149 ± 0.095	1.078 ± 0.079
Protein–ligand contacts (<0.6 nm)	2320 ± 245	1660 ± 168
Average H-bonds	1.51	0.59
Frames with ≥1 H-bond (%)	95.9	45.4
MM-GBSA ΔG (kcal/mol)	−24.96 ± 0.50	−17.81 ± 0.22

1. Molecular Interaction Analysis and Visualization of Protein-Ligand Docking Using Biovia Discovery Studio Visualizer. IJCB 2023, 2, 22, doi:10.24198/ijcb.v2i1.46322.

2. Bell, E.W.; Zhang, Y. DockRMSD: An Open-Source Tool for Atom Mapping and RMSD Calculation of Symmetric Molecules through Graph Isomorphism. J Cheminform 2019, 11, 40, doi:10.1186/s13321-019-0362-7.

3. Warren, G.L.; Andrews, C.W.; Capelli, A.-M.; Clarke, B.; LaLonde, J.; Lambert, M.H.; Lindvall, M.; Nevins, N.; Semus, S.F.; Senger, S.; et al. A Critical Assessment of Docking Programs and Scoring Functions. J. Med. Chem. 2006, 49, 5912–5931, doi:10.1021/jm050362n.
